# Toward semi-automatic biologically effective dose treatment plan optimisation for Gamma Knife radiosurgery

**DOI:** 10.1088/1361-6560/ac8965

**Published:** 2022-10-17

**Authors:** Thomas Klinge, Hugues Talbot, Ian Paddick, Sébastien Ourselin, Jamie R McClelland, Marc Modat

**Affiliations:** 1 Wellcome/EPSRC Centre for Interventional and Surgical Sciences (WEISS), Dept. Medical Physics and Biomedical Engineering, University College London, London, United Kingdom; 2 Centre for Medical Image Computing, Dept. Medical Physics and Biomedical Engineering, University College London, London, United Kingdom; 3 School of Biomedical Engineering & Imaging Sciences, King’s College London, London, United Kingdom; 4 CentraleSupélec, Université Paris-Saclay, Inria, Gif-sur-Yvette, France; 5 Queen Square Gamma Knife Centre, National Hospital for Neurology and Neurosurgery, London, United Kingdom

**Keywords:** Gamma Knife, BED, treatment planning, inverse planning, discrete non-convex optimisation

## Abstract

*Objective.* Dose-rate effects in Gamma Knife radiosurgery treatments can lead to varying biologically effective dose (BED) levels for the same physical dose. The non-convex BED model depends on the delivery sequence and creates a non-trivial treatment planning problem. We investigate the feasibility of employing inverse planning methods to generate treatment plans exhibiting desirable BED characteristics using the per iso-centre beam-on times and delivery sequence. *Approach.* We implement two dedicated optimisation algorithms. One approach relies on mixed-integer linear programming (MILP) using a purposely developed convex underestimator for the BED to mitigate local minima issues at the cost of computational complexity. The second approach (local optimisation) is faster and potentially usable in a clinical setting but more prone to local minima issues. It sequentially executes the beam-on time (quasi-Newton method) and sequence optimisation (local search algorithm). We investigate the trade-off between time to convergence and solution quality by evaluating the resulting treatment plans’ objective function values and clinical parameters. We also study the treatment time dependence of the initial and optimised plans using BED_95_ (BED delivered to 95% of the target volume) values. *Main results.* When optimising the beam-on times and delivery sequence, the local optimisation approach converges several orders of magnitude faster than the MILP approach (minutes versus hours–days) while typically reaching within 1.2% (0.02–2.08%) of the final objective function value. The quality parameters of the resulting treatment plans show no meaningful difference between the local and MILP optimisation approaches. The presented optimisation approaches remove the treatment time dependence observed in the original treatment plans, and the chosen objectives successfully promote more conformal treatments. *Significance.* We demonstrate the feasibility of using an inverse planning approach within a reasonable time frame to ensure BED-based objectives are achieved across varying treatment times and highlight the prospect of further improvements in treatment plan quality.

## 1. Introduction

In stereotactic radiosurgery (SRS) treatments with the Gamma Knife (GK), an array of collimated Cobalt-60 sources is used to precisely deliver therapeutic radiation to an intracranial target volume (TV). Since its introduction over 50 years ago, the GK has become a standard delivery method for SRS procedures (Podgorsak *et al* 1989, Schulder and Patil 2008) and the GK unit has undergone significant updates. So far, there have been five commercially available models that introduced significant changes to the treatment delivery (e.g. geometry of the radiation unit, patient positioning system, collimator selection).

Given that treatments are reported in terms of total physical dose, the significant changes in the time-domain of GK treatment delivery are currently not taken into account under the assumption that GK treatments are single fraction acute exposures. However, it has been shown that the typical time frame of GK SRS treatments would allow for repair of sublethal radiation damage, an effect that is known to be exposure time dependent (Hallgren *et al* 2019).

There have been a number of studies that demonstrated how the biologically effective dose (BED) across different patients treated with the GK varies with the treatment time despite being evaluated for the same physical dose levels (Hopewell *et al* 2012, 2013, Millar *et al* 2015). The model used for these publications was initially derived for a generalised fractionated protracted irradiation under consideration of incomplete repair processes (Millar and Canney 1993) and subsequently, a version of this model using two repair- rates was used in an iso-effect fit to extract the model parameters (Pop *et al* 2000). More recent treatment outcome studies demonstrated an improved correlation with BED compared to the physical dose for single iso-centre trigeminal neuralgia (Tuleasca *et al* 2019), and multi iso-centre acromegaly (Graffeo *et al* 2020) and pituitary adenoma (Graffeo *et al* 2021) SRS treatments (using a simplified version of the BED for retrospective analysis (Jones and Hopewell 2018)). In addition, it has recently been demonstrated how the BED will change with the sequence in which the iso-centres are delivered and also due to unscheduled interruptions in treatment delivery (Klinge *et al* 2021). This is due to the fact that the BED model tracks all changes in the in-patient dose-rate distribution throughout the entire treatment delivery, including beam-off periods. Any attempt at BED-based treatment planning will thus have to consider the exact delivery sequence in addition to the conventional treatment planning parameters.

These studies raise the need to investigate the value of BED-based treatment planning, especially given the large potential for variations in treatment time, number of iso-centres and possible collimator combinations enabled with the modern GK Perfexion (PFX) and Icon^6^ 
^6^
For the cohort in this study, treatment times were 18.2–75.3 min using 3–17 iso-centres.. While there are inverse planning tools available for physical dose GK treatment planning (Sjölund *et al* 2019), they typically rely on formulating a convex treatment planning problem that can be solved efficiently (Levivier *et al* 2018). Using a BED model that incorporates incomplete repair intervals adds a layer of complexity to the problem since the induced radiation damage of an individual iso-centre is linked to the properties of the other iso-centres and can not be treated independently. The nonlinear and non-convex nature of the BED model makes it inherently a hard problem to solve to optimality.

The goal of this study is to explore the value and feasibility of using inverse planning to create BED-based GK treatment plans via adjustments to the beam-on times and delivery sequence (or shot order) using both local and global optimisation techniques. Starting from a plan manually generated using the conventional approach relying only on physical dose, we optimise the original treatment plan in terms of BED by changing the sequence and exposure times only (fixed iso-centre locations and collimator settings). Local optimisation approaches are used to quickly solve for the most beneficial beam-on times (gradient-based (Byrd *et al* 1995)) and delivery sequence (local search (Johnson and McGeoch 1997)). While they are fast, the non-convexity of the BED means that there is also the risk of getting ‘stuck’ in local minima, which in turn can result in less optimal treatment plans. To tackle the problem of non-convexity, a mixed-integer programming (MIP) approach that can simultaneously solve the discrete delivery sequence and continuous per iso-centre beam-on times was developed. This is realised using a convex hull of the BED model to create a mixed-integer linear programming (MILP) problem^7^ 
^7^
The interested reader is referred to Nocedal and Wright (2006) for a general introduction to optimisation problems (e.g. convexity, continuous optimisation, complexity), to Chen *et al* (2009) for an overview of MIP problems, and to Burer and Letchford (2012) for a more specific review of non-convex MINLP programming (e.g. convex hull). The derivation of the MILP approach using the convex hull of the BED is described in the appendix C.. While this approach does consider the entire solution space, the global optimum of such a non-convex function can only be guaranteed with global solvers and (possibly) infinite time. This approach is a trade-off between considering the vast solution space and converging within non-infinite time.

Both optimisation strategies are applied to a cohort of vestibular schwannoma treatments, 14 cases in total, using the original physical dose plans as the starting point. The performances of these approaches are evaluated in terms of their final objective function values, the quality of the optimised treatment plans, and the required time to convergence to determine the clinical feasibility and quality of the individual approaches.

## 2. Method

The proposed semi-automatic creation of BED-optimised treatment plans consists of two steps: iso-centre setup and optimisation. In the initial setup step, the location and collimator settings of the iso-centres are defined. This could be done by conventional (manual) physical dose treatment planning, an initial ‘filling’ step or any physical dose inverse planning module. After this initial phase, we optimise the per iso-centre beam-on times and the sequence of delivery.

### 2.1. Treatment planning problem

#### 2.1.1. BED model

The BED model used in the current study was originally developed by Millar and Canney (1993) as an extension of the linear-quadratic model (Fowler 1989) and refined by Pop *et al* (2000) to include two repair-rates and determine the *α*/*β* ratio (Fowler 1989) for central nervous system tissue. The per voxel BED can be determined as follows:

\begin{eqnarray*}\mathrm{BED}={D}_{T}+\displaystyle \frac{1}{\tfrac{\alpha }{\beta }}\left[\displaystyle \frac{{\mathrm{\Phi }}({\mathrm{\Xi }},{\mu }_{1})+c\cdot {\mathrm{\Phi }}({\mathrm{\Xi }},{\mu }_{2})}{1+c}\right]\sum _{n=1}^{N}{d}_{n}^{2}\end{eqnarray*}

\begin{eqnarray*}{\mathrm{\Phi }}({\mathrm{\Xi }},\mu )=\displaystyle \frac{\tfrac{2}{\mu }\sum _{j=1}^{N}\left[{d}_{j}^{2}\tfrac{\left[\delta {t}_{j}-\tfrac{1}{\mu }\left(1-{e}^{-\mu \delta {t}_{j}}\right)\right]}{\delta {t}_{j}^{2}}-\tfrac{1}{\mu }\sum _{k=1}^{j-1}{d}_{k}{d}_{j}\tfrac{{e}^{-\mu ({t}_{j}-{t}_{k})}({e}^{\mu \delta {t}_{k}}-1)({e}^{-\mu \delta {t}_{j}}-1)}{\delta {t}_{k}\delta {t}_{j}}\right]}{{\sum }_{n=1}^{N}{d}_{n}^{2}},\end{eqnarray*}

with the number of iso-centres *N*, the total dose *D*_*T*_, per iso-centre dose *d*, per iso-centre beam-on time *δ* *t*, start time of the iso-centres *t*, repair-rate *μ*, partition coefficient *c* ^8^ 
^8^
The partition coefficient determines the relative contributions from the two repair-rates., and treatment protocol Ξ describing the dose-rate time dependence. The model parameters as determined by Pop *et al* are shown in table 4 in the appendix. Expressed in terms of the per iso-centre dose-rate $\dot{d}$, the BED equations become:

\begin{eqnarray*}\mathrm{BED}=\sum _{j=1}^{N}\dot{{d}_{j}}\delta {t}_{j}+\displaystyle \frac{1}{\tfrac{\alpha }{\beta }}\left[\displaystyle \frac{{\mathrm{\Psi }}({\mathrm{\Xi }},{\mu }_{1})+c\cdot {\mathrm{\Psi }}({\mathrm{\Xi }},{\mu }_{2})}{1+c}\right]\end{eqnarray*}

\begin{eqnarray*}{\mathrm{\Psi }}({\mathrm{\Xi }},\mu )=\displaystyle \frac{2}{\mu }\sum _{j=1}^{N}\left[{\dot{{d}_{j}}}^{2}\left[\delta {t}_{j}-\displaystyle \frac{1}{\mu }\left(1-{e}^{-\mu \delta {t}_{j}}\right)\right]-\displaystyle \frac{1}{\mu }\sum _{k=1}^{j-1}\dot{{d}_{k}}\dot{{d}_{j}}{e}^{-\mu ({t}_{j}-{t}_{k})}({e}^{\mu \delta {t}_{k}}-1)({e}^{-\mu \delta {t}_{j}}-1)\right].\end{eqnarray*}

The model determines the BED-based on the dose-rate profile throughout the treatment. Each individual iso-centre delivers the dose at a constant dose-rate for a period of time. Practically, this means the iso-centres become individual fractions that are defined by a start time, beam-on time and dose-rate. This formulation implicitly includes periods of beam-off time in-between the delivery of iso-centres where the patient is repositioned and a new set of collimators can be selected. For every iso-centre, all preceding iso-centres are taken into account to determine the residual sub-lethal radiation damage (see nested sum in equation (4)). Consequently, changing the order of delivery will change the BED formulation in two ways: firstly, the nested sum now has to be evaluated over the new sequence, and secondly the starting times of the individual iso-centres need to be updated. Even with only the beam-on times as a variable, the BED (*δ* ***t***) constitutes a non-convex function (i.e. Hessian matrix not positive semi-definite for all *δ* *t*).

#### 2.1.2. Problem definition

After the iso-centre definition (location and shape), the individual per iso-centre dose-rate distributions are fixed, leaving both the per iso-centre beam-on times and the sequence of delivery as free variables. The treatment planning problem using a BED-based objective function *f*(BED) can then be described as follows:

\begin{eqnarray*}\begin{array}{l}\begin{array}{llll}\mathop{\arg \,\min }\limits_{\delta {t}_{j},{seq}} &amp; f(\mathrm{BED}) &amp; &amp; \\ \mathrm{subject}\,\mathrm{to}: &amp; \delta {t}_{j}\geqslant 0, &amp; \delta {t}_{j}\in {\mathbb{R}}, &amp; j\in \{1...N\}\\ &amp; {seq}\in {{\mathfrak{S}}}_{N} &amp; &amp; \end{array}.\end{array}\end{eqnarray*}

The *δ* *t*_*j*_ are the beam-on times of the individual iso-centres *j* and *seq* is their delivery sequence which is constrained to be a member of the symmetric group ${{\mathfrak{S}}}_{N}$ on the finite set {1,.., *N*} of *N* iso-centres. This group is comprised of all *N* *!* possible permutations of the delivery sequence. Due to the characteristics of the BED model and the discrete nature of the sequence, this constitutes a non-convex mixed-integer nonlinear programming problem (MINLP).

#### 2.1.3. Objective function

All optimisation approaches in this study (MILP, gradient-based methods, combinatorial optimisation) use the same objective in order to enable comparisons across methods. The chosen objective function is comprised of the weighted sum of two objectives: the mean BED under-exposure inside the target volume (TV) and the mean BED over-exposure in the normal tissue (NT) around the target (Rim)

\begin{eqnarray*}f({\bf{BED}})={w}_{TV}\displaystyle \sum _{v\in TV}\frac{{\max }(0,{\mathrm{BED}}_{{ref}}-{\mathrm{BED}}_{v})}{{N}_{TV}}+{w}_{Rim}\displaystyle \sum _{v\in Rim}\frac{{\max }(0,{\mathrm{BED}}_{v}-{\mathrm{BED}}_{{thres}})}{{N}_{Rim}}.\end{eqnarray*}

Depending on whether a given voxel is part of the TV or the Rim, its *BED*_*v*_ will be compared to the prescription value *BED*_*ref*_ or the upper permitted threshold *BED*_*thres*_ and the result scaled according to the size of the volume of interest (VOI) (*N*_*TV*_, *N*_*Rim*_). The weighting factors *w* can be used to focus the optimisation more on target coverage or selectivity.

#### 2.1.4. Solving the treatment planning problem

While a brute force approach could theoretically be employed to identify the most beneficial delivery sequence of a given treatment plan, the fact that the number of possible sequences increases with the factorial of the iso-centre number makes this approach impractical for all but the simplest treatments. The first naive way of tackling this problem is to explore efficient and established local approaches to individually optimise the beam-on time (gradient-based) and the delivery sequence (local search). See section 2.2 for a description of the algorithms used in this study.

However, due to the non-convex nature of the BED, there is the possibility for the optimiser to get stuck in a local minimum and never reach the globally optimal value. Ideally, one would like to simultaneously explore all possible combinations of beam-on times and delivery sequences to avoid reaching convergence at a sub-optimal solution. While there are global optimisation techniques, the nonlinear non-convex nature of the BED together with the mixed-integer variables and the large space of possible permutations of the delivery sequence makes solving this problem to optimality prohibitively expensive in terms of computing power and time.

To tackle both problems mentioned above, a ‘convex mixed-integer underestimator’ of the ‘full BED’ was developed (see section 2.3). This reduces the problem to a mixed-integer linear programming problem (MILP). While this convex MILP approach can still not guarantee global optimality, it does consider the entire search space during optimisation and allows to simultaneously optimise the beam-on time and delivery sequence.

### 2.2. Local optimisation approaches

Two established algorithms (see 2.2.1 and 2.2.2) are used to optimise the beam-on time and delivery sequence independently. To achieve the best possible plan with these local approaches, they are executed in an alternating fashion until no further improvement of the objective function is observed.

#### 2.2.1. Beam-on time optimisation

To find the most beneficial set of beam-on times, given the defined objectives and constraints, the limited-memory Broyden–Fletcher–Goldfarb–Shanno bound-constrained (L-BFGS-B) algorithm (Byrd *et al* 1995) for the optimisation of nonlinear problems is used. The L-BFGS-B algorithm utilises the gradient and an approximation of the Hessian (2nd derivative) to guide the direction of the optimisation. For this study the implementation of the scipy.optimize (Virtanen *et al* 2020) library was used with the BED-based objective function as an input. The gradient is approximated numerically during the optimisation.

#### 2.2.2. Delivery sequence

In order to optimise the sequence of delivery, the treatment planning problem is expressed as a travelling salesperson problem (TSP). Instead of the travelling distance, the BED-based objective function is evaluated to determine the quality of a given sequence. To solve this TSP, a common local search algorithm, the 2-opt approach (Croes 1958, Johnson and McGeoch 1997), is adapted to the BED treatment planning scenario. Generally, the 2-opt approach starts with an initial solution, creates a new connection between 2 nodes on the route and solves the order in which all the other nodes will now be visited to create a new, potentially improved solution. Since in our case, there is no distance measure to identify the potential for improved routing (e.g. longest distances, cross-over), the nodes (iso-centres) to be connected (delivered one after the other) are determined iteratively. Starting from the first iso-centre, the objective function is evaluated for which iso-centre should be delivered next (3,…, *N*) until an improvement is found. If no improvement is found, the next iso-centre in the current sequence is chosen as a candidate to be connected to the others. If an improvement was found, the algorithm is started from the beginning. The algorithm is stopped when no more improvement is found for an entire iteration over all iso-centres. Since a treatment delivery is not a closed loop, this approach never changes the starting iso-centre. To mitigate this issue, the 2-opt algorithm is executed once for every iso-centre in the starting position. The initial solution is always created from the current best solution.

### 2.3. Convex mixed-integer underestimator approach (Convex MILP)

In general, MINLP problems are difficult to solve since even their continuous relaxation constitutes a non-convex problem that requires a global optimisation approach to solve. To mitigate this issue, we propose the use of a convex relaxation of the full BED model that can be solved to optimality with available MILP solvers. The ‘relaxed BED’ can then be used as an underestimator for the full problem.

Specifically, the ‘relaxed BED’ will be defined by the bounds on the beam-on times *δ* *t*_*j*_ of the individual iso-centres *j*. When the bounds are initially loose, a large range of beam-on times is permitted for all iso-centres and optimising it returns a lower bound of the feasible objective function value for the ‘full BED’. By iteratively tightening the bounds of the underestimator around its’ current optimum, we can minimise the objective function value (of the ‘full BED’) until the full model and its’ relaxation converge towards each other. For this study, CPLEX 12.10^9^ 
^9^

ILOG CPLEX Optimization Studio V12.10.0 User’s Manual, International Business Machines Corporation, 2021. was used as the MILP solver.

#### 2.3.1. Defining the convex hull of the BED model

The first step in the creation of the ‘relaxed’ BED model is to substitute all nonlinear terms in equation (4) (i.e. all terms including the exponential function) with a new variable. In our case (constant dose-rate), we are left with linear and trilinear terms for the intra and inter iso-centre interactions, respectively.

In order to achieve a convex relaxation, the dual envelope method for general multilinear terms, as described by Costa and Liberti (2012), is applied. The convex hull is defined from the support points created by the combinations of the lower and upper bounds of the variables. Applied to the BED model, these bounds are determined by the values of the substituted nonlinear terms at the lower and upper bounds of the beam-on times *δ* *t*. The Lagrange multipliers that are used to navigate the convex hull have now become the new decision variables.

To constrain the optimisation to physically deliverable treatment plans, the timing information, i.e. beam-on times and starting times of the individual iso-centres, has to be recovered from the nonlinear terms This is achieved by applying a piecewise linearisation (PWL) (Lin *et al* 2013) to each individual substituted term.

In this manner, convex hulls are created for every addend of the nested sum in the full BED model (equation (4)). In total, there are $\tfrac{N(N-1)}{2}$ convex hulls of the interaction terms for a given delivery sequence.

Due to the limitation of the optimisation variables to beam-on times and sequence, the dual envelope formulation of ‘outer sum’ terms is effectively a linearisation. However, the presented approach could still be applied if the dose-rate were to be included as a variable (due to its’ dependence on iso-centre location and collimator selection). The approach would then create a convex hull equivalent to the McCormick envelopes (bilinear). Using this general approach for multilinear functions allows for great flexibility for future expansion of the model.

#### 2.3.2. Delivery sequence

If the delivery sequence can change, then every iso-centre could feasibly interact with all other iso-centres.

To accommodate all possible delivery sequences, the model is extended to include all *N*(*N* − 1) interaction terms with a binary variable (*h*_*jk*_) signifying whether the term is active or not, based on the variable start times (*t*_*j*_, *t*_*k*_) of the iso-centres

\begin{eqnarray*}\displaystyle \begin{array}{rcl}{\mathrm{\Psi }}({\mathrm{\Xi }},\mu ) &amp; = &amp; \displaystyle \frac{2}{\mu }\sum _{j=1}^{N}{\dot{{d}_{j}}}^{2}\left[\delta {t}_{j}-\displaystyle \frac{1}{\mu }\left(1-{e}^{-\mu \delta {t}_{j}}\right)\right]\\ &amp; &amp; -\displaystyle \frac{2}{{\mu }^{2}}\sum _{j\ne k}{h}_{{jk}}\dot{{d}_{k}}\dot{{d}_{j}}{e}^{-\mu ({t}_{j}-{t}_{k})}({e}^{\mu \delta {t}_{k}}-1)({e}^{-\mu \delta {t}_{j}}-1)\end{array}\end{eqnarray*}

\begin{eqnarray*}{h}_{{jk}}=\left\{\begin{array}{lll}1, &amp; \mathrm{if} &amp; {t}_{j}(\delta {\boldsymbol{t}},{seq})> {t}_{k}(\delta {\boldsymbol{t}},{seq})\\ 0, &amp; \mathrm{if} &amp; {t}_{j}(\delta {\boldsymbol{t}},{seq})\leqslant {t}_{k}(\delta {\boldsymbol{t}},{seq})\end{array}\right..\end{eqnarray*}

Using a set of logical constraints, we can ensure that only the appropriate terms describing a feasible delivery sequence are active.

Since the time in-between the beginning of the delivery of two iso-centres (*t*_*j*_ − *t*_*k*_) depends on the specific sequence, its’ bounds need to accommodate this and do not shrink with the beam-on time bounds anymore. As a result, the number of support points of the PWL of the interaction term needs to be increased to ensure that the ‘relaxed’ BED will be close to the ‘full’ BED when the beam-on time is tightened, leading to a more complex optimisation problem. The number of PWL support points is a parameter of the optimisation problem that allows to control the trade-off between model complexity and underestimator accuracy.

### 2.4. Workflow

#### 2.4.1. Data import

The cohort for this study consists of 14 cases of vestibular schwannoma treated with the GK PFX. An overview is shown in table 1. All cases were planned with a prescription dose of 12 Gy and a varying prescription iso-dose. Treatment times ranged from 18 to 75 min, assuming a gap of 0.06 min between iso-centre deliveries.

**Table 1.** Overview of patients treated for vestibular schwannoma with the GK PFX. Listed are the case number, the prescribed dose, the prescription iso-dose, the treatment time *T* (including beam-off periods), the number of iso-centres *N*_*iso*_, the reference dose-rate on the day of treatment, and the TV.

Case	Pres.	Pres.	*T* [min]	*N*_*iso*_	Reference	TV [cm^3^]
	Dose [Gy]	Iso-dose [%]			Dose-rate [Gy min^−1^]	
1	12	52	44.7	4	1.7	0.2
2	12	51	32.4	9	2.4	3.6
3	12	50	46.2	17	2.1	5.1
4	12	43	62.7	16	1.9	12.1
5	12	44	61.2	11	1.7	2.5
6	12	46	75.3	11	1.7	4.7
7	12	44	48.3	12	1.7	5.9
8	12	50	74.8	17	1.6	1.5
9	12	60	28.1	12	3.4	1.8
10	12	58	18.2	3	3.1	0.1
11	12	50	37.3	12	2.8	3.1
12	12	50	58.1	17	2.1	7.0
13	12	42	51.4	12	1.9	4.6
14	12	46	45.3	11	1.9	1.3

TV: Target Volume

To load the previously created treatment plans into the BED treatment planning framework, a research version of GammaPlan 10.1 is used that allows exporting per-isocentre dose distributions. The exported data includes the dose distributions in a 31 × 31 × 31 voxel grid covering the target volume (TV), a binary mask of the TV, the original iso-centre setup (shape, beam-on times and delivery sequence), and a registration matrix to the imaging study.

With this information, the original dose and BED distributions can be calculated and later compared to the optimised versions.

#### 2.4.2. Problem setup

Since using all voxels (almost 30 000) for the optimisation is impractical and would include regions with very limited dose, the problem is constrained to a region of interest (ROI) where a meaningful dose contribution can occur. To this end, a Rim structure is created, which can also drive the optimisation towards normal tissue (NT) sparing. This is achieved by applying 4 iterations of binary dilation to the TV mask using the scipy.ndimage.morphology library from Virtanen *et al* (2020), which effectively grows the TV outwards by 4 voxels. This approach ensures that the number of voxels is approximatively split into a ratio of 60% Rim and 40% TV for all cases of the cohort.

For the present study, both the weights and reference/threshold BED are chosen to be equivalent (${w}_{{TV}}={w}_{{Rim}}=100$, see equation (6)). A treatment time of 60 min is taken as a reference for the BED, as suggested by Jones and Hopewell (2018). With a prescription dose of 12 Gy, this corresponds to a BED of 53.95 Gy_2.47_.^10^ 
^10^
Gy_2.47_ is used to signify BED instead of physical dose (Gy). The subscript denotes the ^*α*^/*β*-ratio used to determine the BED. ^*α*^/*β*-ratio and other BED parameters are shown in table 4 (derived by Pop *et al* 2000). Using the same objective function for all optimisation runs allows for comparing the performance of the different approaches taken to optimise the problem.

#### 2.4.3. Optimisation Runs

We apply the two introduced approaches (local and MILP) to every case in the cohort. For each of the approaches, beam-on time optimisations with and without sequencing (BO-Seq-opt/BO-opt) are executed on a computing cluster using the same setup. Hence, there are four individual optimisation runs per patient. The initial beam-on time is set to 1 min for every case, to avoid accidentally starting from a near-optimal position, e.g. where the original treatment plan was already close to the chosen BED reference.

Due to the large combinatorial complexity for the MILP BO-Seq-opt scenario, a hard limit for the maximum amount of computation time spent in a single iteration is set (deterministic time limit 20e6 ticks corresponds to 20.8–36.8 h^11^ 
^11^
Deterministic time ticks will be the same for repeated solves even with different loads on the system slowing down the ‘real-world’ optimisation time (seconds/hours).) in order to ensure convergence within a reasonable amount of time. If that time limit is exceeded, the currently best solution will be used to update the model for the next iteration. To ensure a feasible solution is found in every iteration, a pre-solving step is executed (i.e. beam-on time optimisation with a fixed sequence). Further details on the parameters chosen for the different optimisation runs can be seen in table 5 in the appendix.

#### 2.4.4. Plan quality indices

The indices describing the treatment plan quality used in this study are defined using following volume ratios:

\begin{eqnarray*}\mathrm{Coverage}:\qquad \qquad C=\displaystyle \frac{V\left({PIV}\cap {TV}\right)}{V\left({TV}\right).}\end{eqnarray*}

\begin{eqnarray*}\mathrm{Selectivity}:\qquad \qquad S=\displaystyle \frac{V\left({PIV}\cap {TV}\right)}{V\left({PIV}\right)}\end{eqnarray*}

\begin{eqnarray*}\mathrm{Paddick}\,\mathrm{Conformity}\,\mathrm{Index}:\qquad \qquad {PCI}=\displaystyle \frac{V{\left({PIV}\cap {TV}\right)}^{2}}{V\left({PIV}\right)\times V\left({TV}\right)}.\end{eqnarray*}

The coverage describes the fraction of the TV that is covered by the prescription iso-dose volume (PIV), while the selectivity describes the fraction of the PIV that is inside the TV. The PCI (Paddick 2000) combines the two proportions to give a measure of conformity. For clinical considerations, it is commonly essential to maintain a particular coverage. Providing the individual values gives the treatment planner additional information (i.e. conformity and how it is ‘split’ between coverage and selectivity). The *D*_95_ and BED_95_ are defined as the minimum dose and BED values delivered to 95% of the TV.

#### 2.4.5. Analysis

The final objective function values are compared across the entire cohort in order to measure the performance of the individual approaches against each other and determine the benefit of explicitly optimising the sequence over using the beam-on times to compensate for the fixed sequence. The clinical scores for coverage, selectivity and PCI are used to determine the initial plan quality in terms of dose and BED, the benefit of the optimisation methods and how well they correlate to the objective function values. Part of the motivation for the BED optimisation is to not only reduce the intra-patient variability but also the inter-patient variability. To visualise this, the original *D*_95_ and BED_95_ values in relation to the treatment time are compared to the ones obtained from the optimised treatment plans. In addition (BE)DVHs and dose/BED distributions of a selection of patients are used to qualitatively describe general changes introduced by the optimisations.

## 3. Results

In this section, the performance of the four different optimisation runs in terms of the minimisation of the objective function (see section 3.1), the resulting quality parameters of the treatment plans (see section 3.2), and examples of the BED-Volume histograms (BEDVHs) and dose/BED distributions (see section 3.3) are presented.

For four of the cases (03, 04, 08, 12) the MILP BED optimisation approach with sequencing did not fully converge, i.e. in the individual iterations of solving the convex underestimator problem no integer-optimal solution was found within the given limit on the computation time^12^ 
^12^
Optimisation parameters ensure a result within approximately 14 d when running on a cluster using 48 threads.. Thus, the current best integer solution is returned and used to update the beam-on time. When optimising the sequence of delivery, the complexity of the problem drastically increases with the number of iso-centres which for these cases was especially large (16 and 17 compared to the maximum of 12 for the rest of the cohort. A plan with 12 iso-centres yields 12*!* = 4.8*e*8 possible sequences, while with 17 iso-centres this number increases to 17*!* = 3.6*e*14). Since these cases are not optimised to completion, they will be excluded from the analysis when comparing the individual optimisation approaches to not skew the results for this optimisation method.

### 3.1. Performance of the optimisation approaches

**Figure 1.**
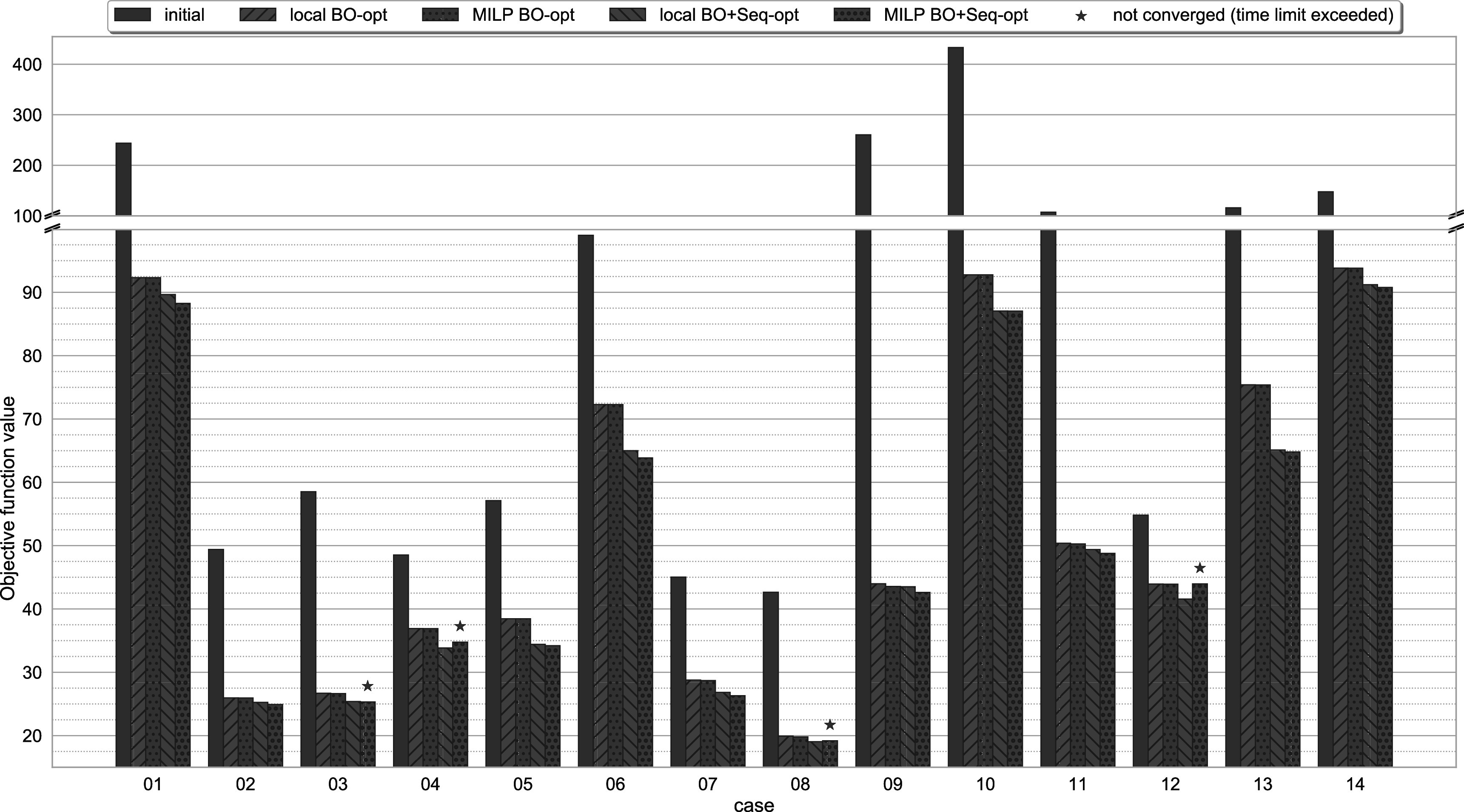
Objective function values for the original treatment plan and the four optimisation scenarios using the local and MILP approaches with and without sequencing. Adding sequencing to the optimisation leads to clear improvements, where the local optimisation reaches close to the performance of the MILP (excluding cases that exceeded the time limit).

The final objective function values are used to assess the performance of the different optimisers. Figure 1 shows an overview of the objective function value for the original treatments compared to the four different optimisation runs. There are large variations for the initial objective function values and all cases in the cohort exhibit significant improvements when using any of the optimisation approaches. Post-optimisation, the objective function values are improved by a factor of approximately 1.25–6.10.

Examining the different optimisation regiments more closely, it becomes evident that the use of the delivery sequence as a variable for the optimisation unlocks the potential for further improvements of the objective function compared to optimising only the beam-on time. Using the local beam-on time optimisation as a reference, the average improvement in objective function value when using sequencing in the optimisation is around 5.86% and 6.95% for the local and MILP optimisations, respectively (see objective function values in table 2).

**Table 2.** Performance Overview: shown are the number of iso-centres *N*_*iso*_, the time *T* to reach convergence, the final objective function value and the relative difference to the baseline of a local beam-on time optimisation.

		Initial		Beam-on time optimisation					Beam-on time + Sequence optimisation					
				Local		MILP			local			MILP		
Case	Niso	obj.	Δ_*obj*_ [%]	*T* [min]	obj.	*T* [min]	obj.	Δ_*obj*_ [%]	*T* [min]	obj.	Δ_*obj*_ [%]	*T* [min]	obj	Δ_*obj*_ [%]
01	4	243.69	164.00	0.01	92.31	0.14	92.31	0.00	0.02	89.65	−2.88	0.67	88.25	−4.40
02	9	49.40	90.33	0.07	25.95	0.60	25.93	−0.08	0.58	25.25	−2.72	159.25	24.92	−3.98
03	17	58.52	119.17	0.41	26.70	6.64	26.64	−0.21	24.85	25.39	−4.90	17 469.19^a^	25.32	−5.18
04	16	48.53	31.48	0.45	36.91	3.90	36.90	−0.02	15.67	33.84	−8.33	15 429.26^a^	34.77	−5.78
05	11	57.10	48.45	0.12	38.46	0.61	38.45	−0.03	1.11	34.41	−10.53	6481.12	34.19	−11.10
06	11	99.02	36.99	0.08	72.28	0.63	72.28	0.00	0.92	64.99	−10.08	3956.95	63.84	−11.67
07	12	45.04	56.54	0.12	28.77	1.25	28.69	−0.27	2.42	26.83	−6.75	10 072.48	26.30	−8.58
08	17	42.63	114.15	0.32	19.91	5.41	19.79	−0.60	21.88	19.04	−4.36	19 043.28^a^	19.19	−3.58
09	12	260.18	491.65	0.13	43.98	3.46	43.56	−0.94	4.14	43.50	−1.08	12 085.30	42.62	−3.09
10	3	432.85	366.55	0.01	92.78	0.16	92.77	−0.01	0.02	87.04	−6.18	0.58	87.02	−6.20
11	12	107.27	112.95	0.13	50.37	1.60	50.27	−0.21	1.98	49.39	−1.96	10 039.29	48.79	−3.15
12	17	54.81	24.76	0.35	43.93	6.12	43.92	−0.03	15.70	41.57	−5.39	19 896.36^a^	43.96	0.07
13	12	115.74	53.52	0.15	75.39	1.71	75.38	−0.01	1.40	65.11	−13.64	6650.02	64.80	−14.06
14	11	147.64	57.36	0.10	93.82	1.12	93.81	−0.01	1.12	91.21	−2.78	5567.23	90.77	−3.25

**Mean:**	**11.71**	**125.89**	**126.28**	**0.17**	**52.97**	**2.38**	**52.91**	−**0.17**	**6.56**	**49.80**	−**5.83**	**9060.78**	**49.63**	**-6.00**
w/o^b^	**9.70**	**155.79**	**147.83**	**0.09**	**61.41**	**1.13**	**61.35**	−**0.16**	**1.37**	**57.74**	−**5.86**	**5501.29**	**57.15**	−**6.95**

MILP: mixed-integer linear programming.

^a^ Case did not fully converge within the time limit.

^b^ Mean value excluding cases that did not converge within the time limit.

In comparison, the differences between the local and MILP optimisations are much smaller. When only using the beam-on time optimisation, the optimisation results are similar. For the beam-on time and sequencing runs, the difference is increased but still small (in the order of 1% improvement when using the MILP optimisation). At the same time, the optimisation times increase significantly with the number of iso-centres. For example, the optimisation time for case 09 is considerably increased from 0.1 min for the local beam-on time optimisation to 4.4 min when optimising the sequence as well. With the MILP approach, the optimisation time increases from 3.5 min to over 200 h due to the incredibly large number of possible delivery sequences. The local approach requires only a fraction of that time (^1^/2900) to reach an objective function value within 2.1% of the MILP result.

### 3.2. Treatment plan quality.

**Figure 2.**
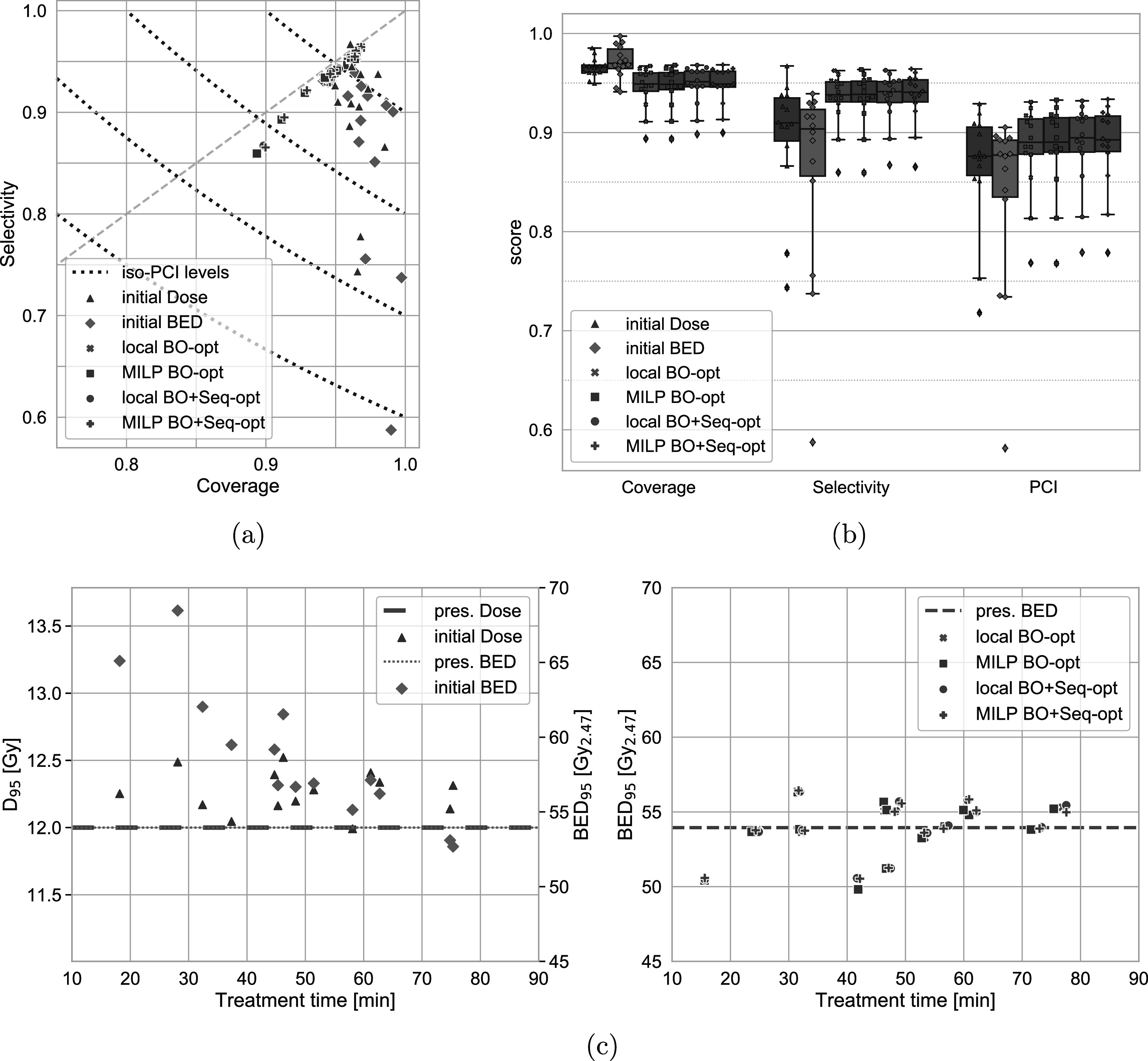
Comparison of the quality parameters of the original and optimised dose and BED distributions. 2(a) Plot of coverage versus selectivity of the individual cases. Initial plans favour coverage over selectivity (more pronounced for BED). Optimised plans balance coverage and selectivity. 2(b) Box plots showing the distribution of quality parameter values for the individual methods. There are only minor variations between the local and MILP optimisations. Improvements, especially in PCI, are more pronounced for sequence optimised plans. 2(c): *D*_95_ and BED_95_ values over the total treatment time. Initial plans show a decrease in BED level with longer treatment times which the physical dose and optimised treatment plans do not exhibit.

To investigate the quality of the optimised treatment plans, the selectivity, coverage, and PCI are evaluated in terms of the prescribed dose (original plans) and prescribed BED (original and optimised plans). The treatment time relationship is assessed using the *D*_95_/BED_95_ values of the treatment plans. Figure 2 gives an overview of the quality parameters for the entire cohort. It can be observed that the original (physical dose) plan favoured coverage (min 95%) over selectivity (min 74%). Evaluating the same original plan in terms of the prescribed BED shows that BED coverage was generally even higher while the selectivity was lower (min 59%). This trade-off at the cost of BED selectivity leads to a lower PCI across the cohort of original treatments.

After optimising these treatment plans, the result shows a slight improvement over the original physical dose PCI. Furthermore, the optimised treatment plans exhibit very similar selectivity and coverage values. Since the generalised objective function used for all cases equally weighs coverage of the TV against sparing of the Rim structure, this behaviour is to be expected.

To distinguish between the different optimisation scenarios, the distribution of the quality parameters (by optimisation method) across the cohort is shown in figure 2(b). Variations across the individual optimisation approaches are small and the only meaningful differences can be observed between optimisations with and without sequencing. Using the sequence optimisation leads to slight improvements in both selectivity and coverage and thus are more pronounced in the PCI values. On average these cases show a PCI of 0.875 compared to 0.872 and 0.815 for the beam-on times only optimisation and original BED, respectively. The optimised PCI values highlight also an improvement over the original dose PCI of .853 on average. Table 3 presents an overview of the mean and range of quality parameters.

**Table 3.** Overview of the objective function values and quality parameters (Selectivity, Coverage, PCI) for the original treatments and the different optimisation scenarios. Shown are the average and range for all cases that were optimised to convergence.

Optimisation		Objective	Selectivity			Coverage			PCI		
		Mean	Mean	Min	Max	Mean	Min	Max	Mean	Min	Max
Orig	Dose	—	0.883	0.744	0.967	0.966	0.952	0.985	0.853	0.718	0.929
	BED	155.79	0.837	0.587	0.930	**0.974**	0.945	0.997	0.815	0.581	0.896

Beam-on	Local	61.41	0.927	0.860	0.962	0.940	0.894	0.967	0.872	0.768	0.931
	**MILP**	61.35	0.927	0.859	0.964	0.940	0.894	0.968	0.872	0.768	0.933

Beam-on	Local	57.74	**0.929**	0.867	0.963	0.942	0.898	0.968	**0.875**	0.779	0.932
and sequence	MILP	57.15	**0.929**	0.865	0.964	0.942	0.900	0.968	**0.876**	0.779	0.934

BED: biologically effective dose, MILP: mixed-integer linear programming, PCI: Paddick conformity index.

One motivation for the use of BED-based treatment plans is to reduce the inter-patient variability due to the variable timings of the dose deliveries (see Jones and Hopewell 2018). The treatment time dependence of the *D*_95_ and BED_95_ values in figure 2(c) exhibits a reduction of the BED with increasing treatment time (52.7–68.5 Gy_2.47_) while there is no time dependence in terms of dose (12–12.5 Gy). After the BED optimisation, the BED_95_ values are distributed around the prescribed BED without any observable treatment time dependence. The range of BED_95_ values across all optimisations is 49.8–56 Gy_2.47_ and is slightly smaller for the optimisations that included the sequencing (beam-on and sequencing: 50.5–56.4 Gy_2.47_, beam-on: 49.8–56.3 Gy_2.47_).

### 3.3. BEDVHs and dose/BED distributions

**Figure 3.**
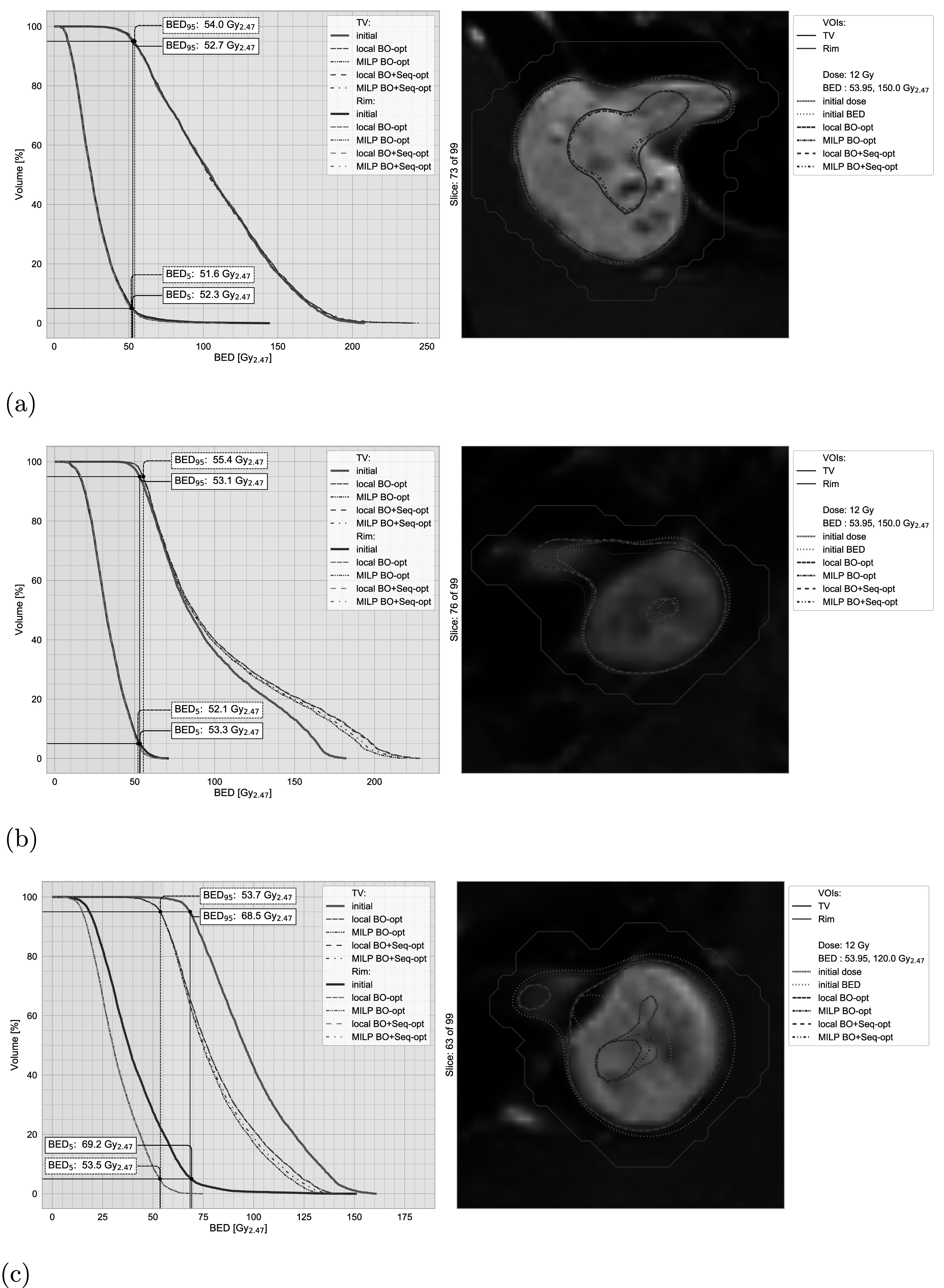
Selected BEDVHs plot with the corresponding dose/BED distributions for the original and BED optimised treatment plans. BED_95/5_ values are marked for the original (solid) and optimised (dashed) treatments. (a) Case 06 exhibits only minor changes after optimisation (mostly limited to the high dose/BED region). (b) Case 08 shows improved conformity of the optimised treatment plans that coincides with an increased high BED region. (c) Case 09 shows that the optimisations lead to a substantial reduction of the BED to the prescribed level.

**Figure 4.**
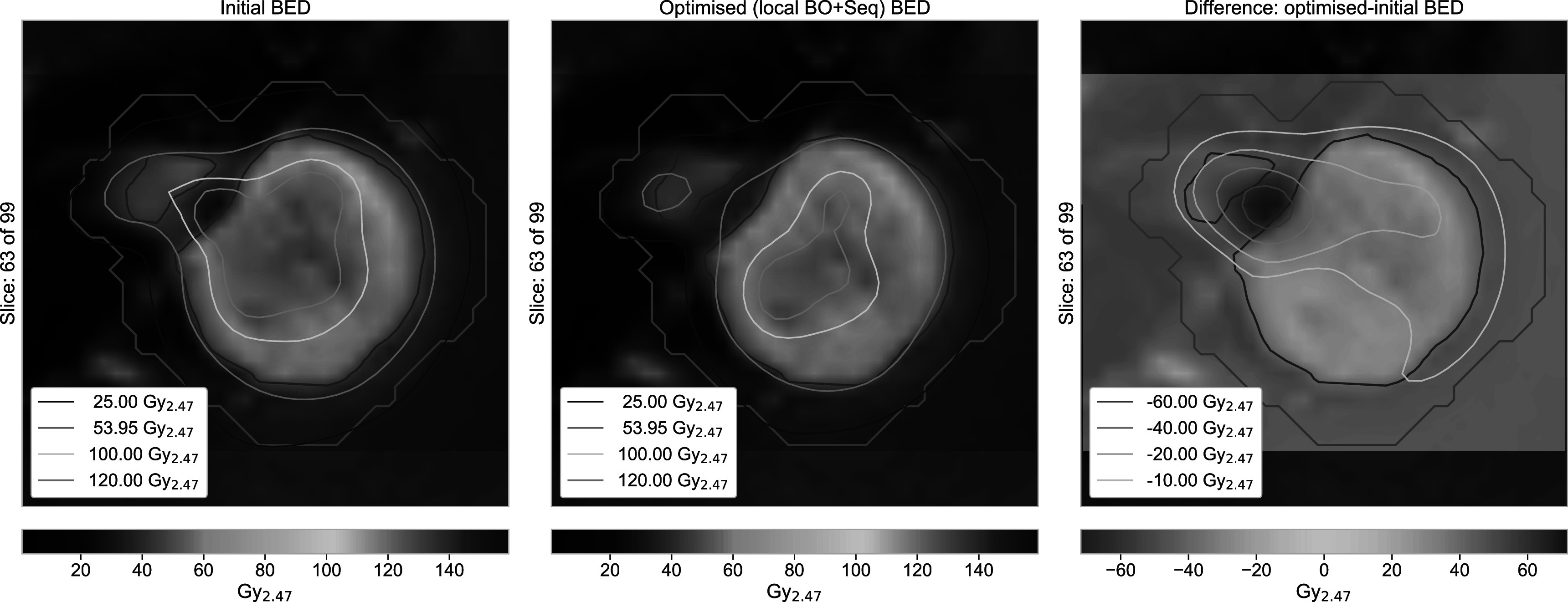
Case 09: BED distributions for the initial and BED optimised (local beam-on time and sequencing) treatment plans showing a substantial BED reduction that is most pronounced in the area of the split in the TV. Negative values in the difference plot indicate a lower BED in the optimised treatment plan. The TV and Rim structures are outlined in red and brown, respectively.

In this section, we present three example cases, one exhibiting only minor changes from the optimisations (case 06), one with improvements to both coverage and selectivity (case 08), and one that revealed substantially higher BED levels for the original treatment (case 09). BEDVHs of the original and optimised treatment plans for the selected cases and the corresponding distributions of the physical dose and BED are shown in figure 3. In addition, figure 4 shows a comparison plot for case 09, visualising the difference in the BED distributions between the initial and locally optimised (beam-on time and sequence) treatment plans.

Several cases happened to be close to the chosen BED reference, mostly due to their original treatment time, and as a result, their BEDVHs exhibit only small changes after the optimisation (see case 06 in figure 3(a)). The minimal changes to the BEDVH are reflected in the similarity of all iso-dose and iso-BED lines for both the original and the optimised treatment plans. Changes in the BED distribution after the optimisations are mostly limited to re-distributions in the high dose/BED region inside of the TV.

Case 08 is an example where both the coverage and selectivity could be improved by the BED optimisation. The BED_95_ of the TV after optimisation is increased by approximately 2.3 Gy_2.47_, while the BED_5_ of the Rim is reduced by approximately 1.2 Gy_2.47_. These improvements appear to be facilitated by a significant increase of BED levels inside the TV where the peak BED is now increased from originally 181 to 216–227 Gy_2.47_ for the optimised treatment plans. The improvements in conformity observed in the BEDVH are also visible in the BED distributions that show improved adherence to the TV shape, especially on the left side (patient right) of the target. This improved conformity of the optimised treatment plans also coincides with an increase in the volume covered by the 150 Gy_2.47_ iso-BED line.

As mentioned before, most cases exhibit marginal BED levels beyond the chosen prescribed BED. An example is case 09 with an originally short treatment time of 28.1 min. Figure 3(c) shows that the volume covered by the prescribed BED is substantially larger than that covered by the prescribed physical dose. After optimisation, the BED is generally scaled down to the reference level (BED_95_ of 53.7 Gy_2.47_). In addition, the split in the target volume in the chosen slice now also exhibits a split in the prescribed BED iso-lines.

The difference plot in figure 4 shows a large area around the split in the TV where the BED is reduced by more than 40 Gy_2.47_, with a peak reduction of 71.8 Gy_2.47_. In the initial treatment plan, large parts of the healthy tissue in the gap of the TV received more than 100 Gy_2.47_ (peak: 140.8 Gy_2.47_).

In contrast to the initial physical dose treatment plans, the proposed optimisation approaches do not enforce a specific heterogeneity in the distributions. The mean prescription iso-dose level across the cohort originally was 48.8% (*σ* = 5.23%) which translates to 25.9% (*σ* = 4.2%) in terms of the reference BED. After optimising the beam-on time and sequence with the MILP approach, these values change to 50.9% (*σ* = 8.3%) and 28.28% (*σ* = 7.4%), respectively.

## 4. Discussion

### 4.1. Optimiser performance

The experiments executed in this study suggest that it is feasible to optimise the per iso-centre beam-on times and the sequence of delivery to create the most beneficial BED distribution. The local approaches do appear to converge to a local minimum, as evident by the fact that the MILP optimisation approach usually converges to a lower objective function value. However, this local minimum appears to be close to the global optimum and no meaningful degradation in plan quality was found as a result of the difference in objective function value. In addition, convergence is reached relatively quickly in the order of seconds/minutes (single-threaded load) compared to hours/days with the MILP approach (multi-threaded).

Using the convex underestimator approach, it is possible to simultaneously consider the beam-on time and order of iso-centre delivery to improve on the local approach. However, the complexity of the MILP models increases dramatically with the number of iso-centres, to the degree that for four of the cases in the cohort (03, 04, 08, 12) with *N*_*iso*_ ≥ 16 the MILP problem could not be solved to integer-optimality within the defined limit on the computation time. Consequently, the next update of the beam-on time bounds is based on the current best solution which can be sub-optimal and lead to the exclusion of the global optimum from the possible solutions. Thus, the final result after a lengthy optimisation (up to 14 d on a compute cluster) can be worse than the local beam-on time optimisation (see case 08). However, given the results for the cases with fewer iso-centres, this is only an issue of computation time and resources and not a flaw of the approach itself. In addition, this approach provides a lower bound for the global optimum within the provided limits on the beam-on time whereas it is impossible to estimate the distance to the global optimum with the local approaches.

### 4.2. Plan quality

Optimising both the beam-on time and sequence can lead to considerably better objective function values, however, this is dependent on the individual cases. In addition, improvements in the objective function value do not necessarily directly translate to increased plan quality.

As mentioned before, most cases exhibit marginal BED levels beyond the chosen prescribed BED. As a result, optimisation typically scales down the BED of the treatment to the appropriate level and increase the selectivity to a similar level of coverage.

The objective function was chosen to be easily compatible across all approaches and create reasonable treatment plans across the entire cohort. It will generally promote both coverage and selectivity to the same degree which results in improved PCI values for the optimised treatment plans. While this relatively simple objective function would be completely minimised (no penalties) for perfect coverage and selectivity, a better objective function value does not directly translate to a better clinical score. In a clinical situation, one would incorporate the planning goals (e.g. minimum 95% coverage, VOI maximum BED thresholds) and quality parameters in the objective function and tailor the weights of the different objectives to the individual patient to achieve the most beneficial treatment plans. The improvements in the overall PCI with this generic objective function indicate that it would be possible to not only reach the same treatment plan quality as for the physical dose plan, but improve on it. It would appear that the added complexity of using a BED model with incomplete repair intervals also provides an additional degree of freedom (in the time domain) that could allow for the creation of even more conformal treatment plans than currently possible.

An example for this is case 08 where both the selectivity and coverage are above the original levels of the physical dose plan. This improvement comes with an increase of the high BED region at the centre of the target. When introducing BED-based treatment plans, one will have to investigate the appropriate prescription BED values and the most beneficial range of BED values inside the TV.

### 4.3. BED prescription

In this study, the prescription BED was chosen according to Jones and Hopewell (2018) with a reference treatment time of 60 min. For the present cohort, this happens to represent a relatively low BED, requiring the optimisation to generally scale down the overall BED level. This is of course only one proposed way to take the treatment time into account and define a reference BED. If a shorter treatment time was chosen as a basis, the present cohort could have appeared to generally exhibit a lower BED than desired. Nevertheless, the results in this study demonstrate that the optimisation approaches can both scale up or down the overall BED level and optimise the conformity of the given cases. Even without enforcing a certain level of heterogeneity to the BED distribution, the optimised treatment plans exhibit similar prescription iso-dose and iso-BED levels as the clinical treatments. This suggests that the inherent heterogeneity in the delivered GK dose is generally maintained when optimising the BED in the presented scenarios.

Additional studies are needed to determine an appropriate prescription BED that signifies a beneficial trade-off between the therapeutic effect and the incidence of adverse effects. An example would be the publication by Tuleasca *et al* (2019) that investigated the treatment of trigeminal neuralgia, which found that increasing BED values above a certain threshold increases the risk of complications without increasing the probability of pain control any further. Accurately modelling the tumour control probability (TCP) and normal tissue complication probability (NTCP) can be a valuable tool for determining the therapeutic window of a given treatment regimen.

### 4.4. Contouring the VOIs

When using inverse planning, the contours that are created determine whether a voxel is considered to be part of the TV, an OAR or the NT. Thus, special care has to be taken to ensure an accurate representation of the treatment planning problem. As can be seen for case 09, where there was a gap in the TV on a single slice (slice thickness: 1.5 mm), the optimiser does not distinguish between voxels of the same class based on their location/surrounding tissue and will adapt the treatment plan accordingly to minimise the objective function. In this case, a lower objective function value is achieved by additional sparing of the NT in this small gap, a characteristic which was not observed in the original treatment plan. This was likely done to ensure coverage of the entire TV. With the inverse planning approach, these high BED values in the NT (regardless of their location) are penalised which leads to the observed BED reductions in that area. This highlights the importance of the drawn contours being consistent with the intention of the treatment planner. If the intention is to accept higher BED values in this gap, an additional contour with a higher BED threshold or lower penalty weight could be added in this location.

In a clinical scenario, where appropriate target coverage is required, the treatment planner would then adapt either the objectives or the contours to ensure the clinical goals are met. In addition, the contouring step will have to include any VOIs in the vicinity of the TV that need to be considered during the treatment plan optimisation. Avoidance of non-delineated critical structures, which a human planner might be aware of during manual planning, is not possible with an inverse planning approach.

### 4.5. Expansion of the treatment planning framework

Having established the local optimisation methods to be a suitable choice for BED treatment planning, the next step would be to include further variables into this optimisation approach to explore the possibility of not only matching the quality of the original dose plan but to improve upon it. One candidate is the iso-centre location. To use the same L-BFGS-B optimisation framework to simultaneously optimise both the beam-on times and the iso-centre locations, some approximations have to be made. Firstly, the shape of the distribution is assumed to remain unchanged for small displacements of a few millimetres. Secondly, to allow for continuous values of the displacement, the dose-rate matrix is linearly interpolated at the candidate locations.

**Figure 5.**
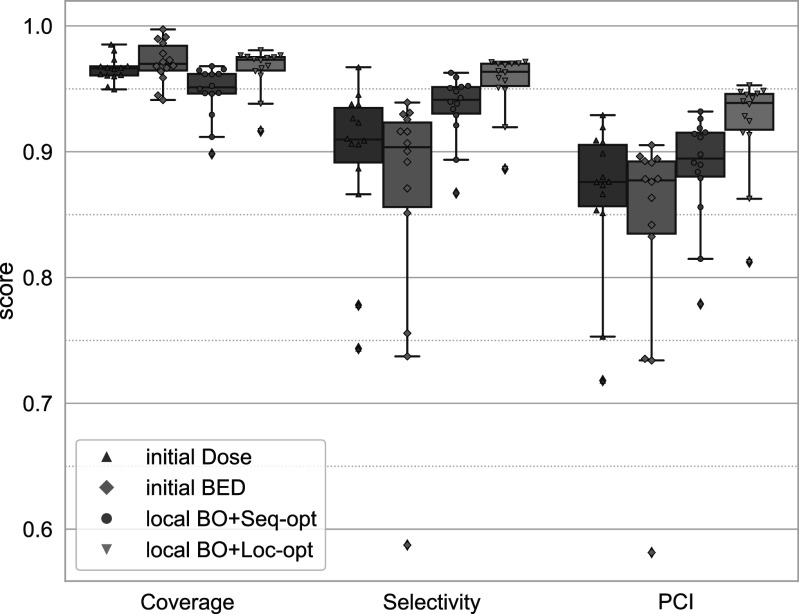
Quality parameters for the original dose and BED distributions and the local optimisation approach using either beam-on time and sequencing or beam-on time and iso-centre location optimisation. Location optimised plans show the potential for improvements beyond the level of the original physical dose treatment plans.

We integrated the iso-centre location optimisation in our proposed framework and figure 5 shows the quality parameters for the original treatment, the local beam-on time and sequencing approach and the location and beam-on time optimisation. Clear improvements, even beyond the values for the original dose treatment plan can be observed. While the mean coverage now matches the original dose value, the mean selectivity is increased from 89.6% to 95.5%. These preliminary results show the potential for meaningful improvements of the treatment plan quality by taking advantage of the added degree of freedom provided by a BED model.

Additionally, the simultaneous optimisation of sequence and beam-on time could be extended to the selection of the most beneficial collimators for the individual sectors. Different combinations of optimisation variables could then be compared to determine the best trade-off between the increased complexity of the optimisation and the expected improvements of the treatment plans.

Having demonstrated the ability for BED-based inverse planning, the next step is to establish a semi-automatic workflow that allows optimising individual treatment plans in a clinical scenario. This would include more practical objectives to individualise treatment plans and a level of interactivity allowing to explore the feasible trade-offs for a given treatment plan. In general, the investigated semi-automatic approach could be fully automated if an initial ‘filling’ step was used for the iso-centre definition as available in Leksell GammaPlan.^13^ 
^13^
See Leksell GammaPlan Online Reference Manual. Article No. 102300 Rev. 01, Elekta Instrument AB Stockholm, Sweden, 2011.


Given a consensus about BED-based prescriptions is found, such an approach could be translated into the clinical workflow. This would require some investment into the software development of the system, e.g. to adapt the treatment planning workflow and tailor the optimisation algorithms to the available hardware. GammaPlan Lightning is the most recent example of a proposed novel inverse planning approach (see Sjölund *et al* 2019) being integrated into the clinical treatment planning system.

The computationally expensive convex underestimator approach could be used as a benchmark to assess other viable optimisation methods. One could create a number of artificial test cases which can be optimised to a very high degree with the MILP approach. Publishing these test cases with the appropriate results could then allow others to benchmark their solutions without having to run the costly optimisation themselves.

### 4.6. The role of BED in GK SRS

While there is evidence that dose-rate effects like those expressed in the BED formulation influence the treatment outcome in terms of the therapeutic effectiveness and incidence of adverse effect, it is not the only measure influencing treatment success and not all studies find a good correlation between the BED and the investigated treatment endpoints.

For trigeminal neuralgia treatments, a study by Tuleasca *et al* (2019) determined a beneficial BED range to minimise the risk of patients developing hypoesthesia without compromising the long term pain-free incidence. Since these were single iso-centre treatments, the BED could be calculated directly from the physical dose without the need to consider interactions across iso-centres which would require access to per iso-centre dose maps.

For multi iso-centre treatments, several BED approximation methods were developed by Jones and Hopewell (2018) that allow for the determination of the marginal BED value from the prescribed dose and treatment time. These simplified BED models do not capture all the information contained in the delivered 3D distributions but allow for a convenient way to retrospectively estimate marginal BED values. The simplified BED was successfully used to determine treatment outcome correlations in acromegaly (Graffeo *et al* 2020) and pituitary adenoma Graffeo *et al* (2021) SRS treatments. The importance of adhering to the model constraints has been highlighted in a letter to the editor by Hopewell *et al* (2021).

There have also been discussions about the role of the reference dose-rate in the context of outcome correlation. One important distinction to make is that the BED used in the presented study is determined based on the 3D in-patient dose-rate distribution while the reference dose-rate value is based on the calibration measurement of the specific GK unit at the centre of a phantom. Factors like collimator size, sector blocking and patient geometry make it infeasible to infer one from the other. Illustrative examples of this are provided in Paddick *et al* (2019), where similar treatment times and BED values are observed for varying reference dose-rates (1–4 Gy min^−1^).

Another factor to consider is the ^*α*^/*β*-ratio. For this study, the value 2.47 Gy, as determined by Pop *et al* (2000) together with the repair-rates and partition coefficient, is used. Analogous to previous studies Hopewell *et al* (2012, 2013), Millar *et al* (2015), Klinge *et al* (2021), this fixed value of the ^*α*^/*β*-ratio is used for the BED calculation. Jones *et al* (2020) report minor effects on equivalent doses for radiation myelopathy in the central nervous system when varying the ^*α*^/*β*-ratio (1.5–3.0 Gy). Using the commonly accepted value of 2 Gy resulted in a <1% deviation of the equivalent single doses for treatment times of up to 5 h. If one were able to extract additional and precise patient-specific information about all involved tissues before the treatment, the likely tissue response could be better quantified. This information could include ^*α*^/*β*-ratios for all involved tissues and additional or various repair-rates. The presented optimisation approaches allow for the assignment of per-voxel tissue parameters and any number of desired repair-rates. The treatment plan could then be tailored to the specific targeted tissues on a per-patient basis. In practice however, this information is not currently available for treatment planning.

There is certainly a need to further investigate all factors that can influence treatment outcomes to provide treatment planners with the tools required to ensure the best possible treatment. One of these tools is the BED. Previous studies have used the BED model to retrospectively investigate variations within the same prescription dose level (Hopewell *et al* 2012, 2013, Millar *et al* 2015, Klinge *et al* 2021) and investigate outcome correlations (discussed above). In contrast, the presented work investigates how these findings could be used to further improve SRS treatments in the future. This requires the development of new optimisation methods to enable the use of the more complex BED model developed by Millar and colleagues for inverse planning. There currently exist no BED treatment planning solutions. This study is an important step towards possible future treatment planning systems.

## 5. Conclusion

This study demonstrates multiple strategies that could be employed to make BED planning feasible in clinical settings. BED treatment planning could significantly reduce the inter-patient variability and has the potential to improve the outcome of GK radiosurgery treatments.

For our cohort, the local optimisation approaches are sufficient to reach the same level of treatment plan quality as the more complex MILP approaches. Furthermore, it is feasible to optimise both the beam-on times and the delivery sequence together to improve on treatment plans created with only the beam-on times as a variable. The MILP approach, while less prone to local minima issues, is too computationally expensive to be used in its current form in a clinical workflow without further significant improvements in optimisation speed. Nevertheless, it can be helpful to provide a lower bound on the achievable objective function value and could be used to benchmark new optimisation approaches.

With the described optimisation methods it is possible to (at least) reach the same treatment plan quality in terms of BED as for the original physical dose treatment plans.

## Appendix A. BED model

Millar and Canney (1993) derived a model to determine the BED of a fractionated, protracted treatment with a variable dose-rate and multiple damage and repair processes. In their work, they extend upon the principle assumptions of the linear-quadratic (LQ) model:

1. The fractional cell death rate is proportional to the rate of induction of lethal damage (*extended to include the effects due to a polynomial representation in the dose-rate*)
2. There are two components of cellular damage: direct lethal damage and sublethal damage
3. The rate of induction of sublethal damage is proportional to the dose-rate (*extended to include the effects due to a polynomial representation in the dose-rate*)
4. Sublethal damage is potentially repairable and is governed by a single exponential repair rate process (*modified to include effects due to multiple types of damages, each of which may be repaired by multiple repair mechanisms*)
5. Certain types of sublethal damage can be converted to lethal damage by further irradiation. Only convertible sublethal damage will be considered.
6. Only a small proportion of induced sublethal damage is converted to lethal damage
7. The rate of production of lethal damage derived from sublethal damage is proportional to both the dose-rate and the amount of sublethal damage (*extended to include the effects due to a polynomial representation in the dose-rate*)

**Table 4.** BED model parameters for *α*/*β*-ratio, repair-rates *μ*_1,2_ and partition coefficient *c* used in this study. The partition coefficient determines the relative contributions from the two repair-rates. A value of 1 represents equal contributions from slow and fast repair. These values were determined by Pop *et al* (2000).

Parameter:	*α*/*β* [Gy]	*μ*_1_ $[\tfrac{1}{\min }]$	*μ*_2_ $[\tfrac{1}{\min }]$	*c*
Value:	2.47	0.0608	0.0053	0.98

These assumptions allow them to describe the time-dependent fractional rate of induced cell death for an arbitrary fractionated protracted treatment. Integration over the duration of the treatment then allows the determination of the biological effect of the entire treatment. The addition of further assumptions that more closely describe a clinical treatment with the GK (or any radiosurgical treatment) allows the simplification of the model. These assumptions are a constant dose-rate during each fraction (i.e. iso-centre), a varying dose-rate level between individual fractions, a single repair rate process, and first-order polynomials for the induction of lethal damage, sublethal damage and translation from sublethal to lethal damage.

This simplification of the model can be formulated as the general LQ model with an additional correction function describing the dose delivery. Pop *et al* (2000) derived a bi-exponential version of this BED model from animal studies and this BED model has since been well established and used by numerous studies over the past decades. As such we refer the reader to the previous work cited in the manuscript for a thorough analysis of the underlying principles.

## Appendix B. BED optimisation settings

**Table 5.** Settings for the different BED optimisation techniques employed in this study.

Scenario	Solver	Settings	Workflow
Original	—	Beam-on times from dose calculation	Calculate BED with original order of iso-centres as sequence
Local	L-BFGS-B	Approx grad: True, bounds: (0, None), *m*: 50	Provide BED objective function and initial beam-on time vector
BO-opt		Epsilon: 1 s, maxiter: 250	Optimise to convergence
Convex MILP	CPLEX MIP	Threads: 48, mip tolerance mipgap: 0.002,	Build underestimator for beam-on of 1 min +/− 10 min − > optimise − >
BO-opt		mip_heuristicfreq: -1, mip_limits_cutpasses: -1	
		Convergence criteria: min bounds: 0.01 min,	Update bounds to *dt*_*opt*_ +/− 60% of last interval − > optimise until convergence
		rel. diff ‘relaxed’ versus full: 2*x* <1*e*-3,	
		individual objective function rel. diff: 4*x* <1*e*-3	
Local	L-BFGS-B	Approx grad: True, bounds: (0, None), *m*: 50,	First execute beam-on optimisation, then optimise sequence
BO+Seq-opt		Epsilon: 1 s, maxiter: 250	
	2-opt	Convergence: obj func rel. diff: 3*x* < 1*e*-3	Alternate to convergence
Convex MILP	CPLEX MIP	Threads: 48, mip tolerance mipgap: 0.002,	Start with beam-on MILP
BO+Seq-opt		mip_heuristicfreq: -1, mip_limits_cutpasses: -1,	Until beam-on time bounds < +/− 1 min
		det time limit: 20e6, mip_limits_repairtries: 300	Switch to model with sequence optimisation
		Convergence criteria: min bounds: 0.01 min,	Update bounds to *dt_opt_* +/− 60% of last interval
		rel. diff ‘relaxed’ versus full: 2*x* <1*e*-3,	Optimise until convergence
		individual objective function rel. diff: 7*x* <1*e*-3	

BED: biologically effective dose, L-BFGS-B: limited-memory Broyden–Fletcher–Goldfarb–Shanno bound-constrained algorithm, MILP: mixed-integer linear programming.

## Appendix C. Creation of the MILP problem

MINLP problems are generally challenging to solve and can require immense computing resources (Burer and Letchford 2012, Köppe 2012). A common solution approach is to find a reformulation of the problem that has beneficial properties like convexity (Burer and Letchford 2012). This study introduces a method that creates a convex hull of the problem to solve it iteratively.

This section covers the steps required to create this convex relaxation (MILP) of the general MINLP treatment planning problem. Firstly, short general descriptions of the necessary methods are presented, and secondly, their application to the BED optimisation problem is described.

**Notation** Throughout the derivation of the MILP formulation, the following conventions are used to describe different objects.

The application of a convex envelope is described by the introduction of a ’ $\hat{}$ ’. So $\hat{w}$ refers to the convex envelope of *w*.

Vector and matrix variables are expressed with bold lettering and their individual scalar components are referenced with subscripts (e.g. all beam-on times *δ* ***t*** with the *j*th beam-on time *δ* *t*_*j*_). For the BED, the subscript index identifying the current voxel is omitted when not specifically required. This is done to aid readability where the BED is completely analogous across all voxels, i.e. the only difference between the voxels *v* is their dose-rate parameter ${\dot{d}}_{{jv}}$ for iso-centre *j*.

### C.1. Convex envelopes

The convex relaxation of the optimisation problem is realised using convex envelopes for multilinear functions *w*(*x*)

\begin{eqnarray*}w(x)={x}_{1}\cdot \ldots \cdot {x}_{k},\qquad \qquad \forall j\leqslant k:\ {x}_{j}\in [{x}_{j}^{L},{x}_{j}^{U}],\qquad \qquad k\in {\mathbb{N}}\end{eqnarray*}

with known lower *x*_*j*_^*L*^ and upper *x*_*j*_^*U*^ bounds of all variables. The multilinear function *w*(*x*) is replaced with a new variable $\hat{w}$ and a set of constraints defining the convex relaxation.

#### C.1.1. McCormic envelopes

If the function is bilinear (*k* = 2), two linear constraints define a lower convex and upper concave envelope. These explicit constraints are known as the McCormick inequalities (McCormick 1976):

\begin{eqnarray*}{convex}:\qquad \qquad \hat{w}\geqslant {x}_{1}^{L}{x}_{2}+{x}_{2}^{L}{x}_{1}-{x}_{1}^{L}{x}_{2}^{L}\end{eqnarray*}

\begin{eqnarray*}\hat{w}\geqslant {x}_{1}^{U}{x}_{2}+{x}_{2}^{U}{x}_{1}-{x}_{1}^{U}{x}_{2}^{U}\end{eqnarray*}

\begin{eqnarray*}{concave}:\qquad \qquad \hat{w}\leqslant {x}_{1}^{L}{x}_{2}+{x}_{2}^{U}{x}_{1}-{x}_{1}^{L}{x}_{2}^{U}\end{eqnarray*}

\begin{eqnarray*}\hat{w}\leqslant {x}_{1}^{U}{x}_{2}+{x}_{2}^{L}{x}_{1}-{x}_{1}^{U}{x}_{2}^{L}.\end{eqnarray*}

These inequalities restrict the values of $({x}_{1},{x}_{2},\hat{w})$ to be within the polyhedron defined by the vertex set of the possible combinations of lower and upper bounds of the variables ${P}_{W}=\{({x}_{1}^{L},{x}_{2}^{L},{x}_{1}^{L}{x}_{2}^{L}),({x}_{1}^{U},{x}_{2}^{U},{x}_{1}^{U}{x}_{2}^{U}),({x}_{1}^{U},{x}_{2}^{U},{x}_{1}^{U}{x}_{2}^{U}),({x}_{1}^{U},{x}_{2}^{L},{x}_{1}^{U}{x}_{2}^{L})\}$.

#### C.1.2. Dual envelopes

In general, convex envelopes of multilinear terms are vertex polyhedral (Rikun 1997, Tardella 2008). To take advantage of this property, Costa and Liberti (2012) propose to express the points inside the envelope as the convex combination of its extreme points ${P}_{W}=\{{p}_{1},\ldots ,{p}_{{2}^{k}}\}\subseteq {{\mathbb{R}}}^{k+1}$. Their approach, named dual envelopes, allows them to define the convex hull of a general multilinear term for an arbitrary value of $k\in {\mathbb{N}}$.

They introduce a vector $\lambda \in {{\mathbb{R}}}_{\geqslant 0}^{{2}^{k}}$ of nonnegative Lagrange multipliers that defines a point such that:

\begin{eqnarray*}x=\sum _{i\geqslant {2}^{k}}{\lambda }_{i}{p}_{i}\qquad \qquad \wedge \qquad \qquad \sum _{i\geqslant {2}^{k}}{\lambda }_{i}=1.\end{eqnarray*}

To create the envelopes, the *p*_*i*_'s need to be expressed as a function of the bounds on the variables *x* ^*L*^, *x* ^*U*^. Costa and Liberti define two parameter sequences, *d*_*ij*_ and *b*_*j*_(*d*_*ij*_). The value of *d*_*ij*_ ∈ {0, 1} describes whether the *j*th component of *p*_*i*_ is a lower or upper bound, while *b*_*j*_(*d*_*ij*_) returns the value of the specific bound:

\begin{eqnarray*}\forall i\leqslant {2}^{k},j\leqslant k\qquad \qquad {d}_{{ij}}=\left(\left\lfloor \displaystyle \frac{i-1}{{2}^{k-j}}\right\rfloor \mathrm{mod}2\right)\end{eqnarray*}

\begin{eqnarray*}\forall j\leqslant k\qquad \qquad {b}_{j}(0)={x}_{j}^{L}\qquad \wedge \qquad {b}_{j}(1)={x}_{j}^{U}.\end{eqnarray*}

The *k*-linear term *w*(*x*) = *x*_1_ ⋯ *x*_*k*_ can then be relaxed with the introduction of 2^*k*^ constrained variables *λ*_*i*_ and *k* + 1 new constraints:

\begin{eqnarray*}\hat{w}=\sum _{i\leqslant {2}^{k}}{\lambda }_{i}\prod _{j\leqslant k}{b}_{j}({d}_{{ij}})\end{eqnarray*}

\begin{eqnarray*}\forall j\leqslant k\qquad {x}_{j}=\sum _{i\leqslant {2}^{k}}{\lambda }_{i}{b}_{j}({d}_{{ij}})\end{eqnarray*}

\begin{eqnarray*}\sum _{i\leqslant {2}^{k}}{\lambda }_{i}=1\qquad \wedge \qquad \forall i\leqslant {2}^{k},{\lambda }_{i}\geqslant 0.\end{eqnarray*}

This relaxation is executed individually for every term in the nested sum of the BED formulation (see equation (4)). In this way, the convex hull of a problem containing *k*-linear terms can be constructed, allowing the relaxed problem to be optimised with the Lagrange multipliers *λ*_*i*_ as the decision variables.

### C.2. Big *M* Method

When formulating the problem, there are situations where the product of two variables needs to be determined. An example is the use of the interaction terms in equation (7), where a binary variable is introduced to determine the active terms To solve the model with the proposed methods, the product of the two involved variables needs to be linearised (Glover 1975). For the multiplication of a bounded continuous variable *M* ∈ [0, *M* ^*U*^] and binary variable *x* ∈ {0, 1}, a new variable *z* = *M* · *x* and a set of constraints can be introduced:

\begin{eqnarray*}z\leqslant {M}^{U}\cdot x\end{eqnarray*}

\begin{eqnarray*}z\leqslant M\end{eqnarray*}

\begin{eqnarray*}z\geqslant M-(1-x)\cdot {M}^{U}\end{eqnarray*}

\begin{eqnarray*}z\geqslant 0.\end{eqnarray*}

These linear inequalities ensure that *z* is within the expected bounds [0, *M* ^*U*^], equal to zero if *x* = 0, and equal to *M* if *x* = 1. This approach is commonly referred to as the big *M* method. Similarly, if the continuous variable is bounded to a non-positive interval [*M* ^*L*^, 0], the variable *z* = *M* · *x* will require the following constraints:

\begin{eqnarray*}z\geqslant {M}^{L}\cdot x\end{eqnarray*}

\begin{eqnarray*}z\geqslant M\end{eqnarray*}

\begin{eqnarray*}z\leqslant M-(1-x)\cdot {M}^{L}\end{eqnarray*}

\begin{eqnarray*}z\leqslant 0.\end{eqnarray*}

For two binary variables *x*, *y* ∈ {0, 1}, we can define *z* = *x* · *y* together with the following three inequalities:

\begin{eqnarray*}z\leqslant x\end{eqnarray*}

\begin{eqnarray*}z\leqslant y\end{eqnarray*}

\begin{eqnarray*}z\geqslant x+y-1.\end{eqnarray*}

These linearisations will be used to facilitate the sequence optimisation that requires the introduction of additional binary variables (see appendix C.4.6).

### C.3. Piece-wise linearisation

To constrain the optimisation to physically deliverable treatment plans, the timing information, i.e. beam-on times and starting times of the individual iso-centres, has to be recovered from the dual envelope formulation without re-introducing nonlinearity to the optimisation. This is achieved by applying a piecewise linearisation (PWL) (Lin *et al* 2013) to each individual substituted nonlinear term.

For a single variable function $f(x):{\mathbb{R}}\to {\mathbb{R}}$ with *x* ∈ [*a*_0_, *a*_*m*_], a set of *m* + 1 monotonically increasing support points {*a*_*l*_ ∣ *l* ∈ {0,…,*m*}, *a*_0_ < *a*_1_ < ⋯ < *a*_*m*_} can be defined. An approximate PWL of *L*(*f*(*x*)) over the interval [*a*_0_, *a*_*m*_] can then be achieved using the two surrounding support points *a*_*l*_ ≤ *x* ≤ *a*_*l*+1_ and their individual weights *ν*_*l*_:

\begin{eqnarray*}L(f(x))=\sum _{l=0}^{m}{a}_{l}{\nu }_{l}\end{eqnarray*}

\begin{eqnarray*}x=\sum _{l=0}^{m}{a}_{l}{\nu }_{l}.\end{eqnarray*}

This requires the use of the following constraints:

\begin{eqnarray*}{\nu }_{0}\leqslant {y}_{0},\qquad {\nu }_{m}\leqslant {y}_{m-1}\end{eqnarray*}

\begin{eqnarray*}{\nu }_{l}\leqslant {y}_{l-1}+{y}_{l},\qquad \mathrm{for}\,l=1,\ldots ,m-1\end{eqnarray*}

\begin{eqnarray*}\sum _{l=0}^{m-1}{y}_{l}=1\end{eqnarray*}

\begin{eqnarray*}\sum _{l=0}^{m}{\nu }_{l}=1\end{eqnarray*}

\begin{eqnarray*}{y}_{l}\in \{0,1\},\qquad {\nu }_{l}\geqslant 0,\qquad \mathrm{for}l=0,\ldots ,m-1.\end{eqnarray*}

The binary variable *y*_*l*_ determines the active interval for any value of *x* ∈ [*a*_0_, *a*_*m*_] and thus which two adjacent *ν*_*l*_ take a nonzero value.

The number of new (binary) variables and linear constraints introduced with this approach scales linearly with the number of support points *m*. A higher number of support points increases both the accuracy of the PWL and the computational cost to solve the problem.

### C.4. Convex relaxation of the MINLP problem

#### C.4.1. Reformulating the BED

To implement the optimisation of the BED, we need to express it in terms of the actual decision variables and parameters used by the solver. Specifically, we define a new variable Δ*t* that describes the difference in starting time of two iso-centres and relate this back to the individual combination of beam-on times *δ* ***t*** and gaps ***g*** between iso-centre deliveries. In addition, we substitute the nonlinear terms and combine constants that are the same across multiple summands into a single parameter. *Original:*

\begin{eqnarray*}\mathrm{BED}=\sum _{j=1}^{N}\dot{{d}_{j}}\delta {t}_{j}+\displaystyle \frac{1}{{}^{\alpha }/\beta }\left[\displaystyle \frac{{\mathrm{\Psi }}({\mathrm{\Xi }},{\mu }_{1})+c\cdot {\mathrm{\Psi }}({\mathrm{\Xi }},{\mu }_{2})}{1+c}\right]\end{eqnarray*}

\begin{eqnarray*}\displaystyle \begin{array}{rcl}{\mathrm{\Psi }}({\mathrm{\Xi }},\mu ) &amp; = &amp; \displaystyle \frac{2}{\mu }\sum _{j=1}^{N}\left[{\dot{{d}_{j}}}^{2}\left[\delta {t}_{j}-\displaystyle \frac{1}{\mu }\left(1-{e}^{-\mu \delta {t}_{j}}\right)\right]\right.\\ &amp; &amp; -\left.\displaystyle \frac{1}{\mu }\sum _{k=1}^{j-1}\dot{{d}_{k}}\dot{{d}_{j}}{e}^{-\mu ({t}_{j}-{t}_{k})}({e}^{\mu \delta {t}_{k}}-1)({e}^{-\mu \delta {t}_{j}}-1)\right].\end{array}\end{eqnarray*}

*Reformulated:*

\begin{eqnarray*}\displaystyle \begin{array}{rcl}\mathrm{BED} &amp; = &amp; \sum _{j=1}^{N}\dot{{d}_{j}}\delta {t}_{j}+{A}_{1}\sum _{j=1}^{N}{\dot{{d}_{j}}}^{2}{w}_{{A}_{1}}(\delta {t}_{j})+{A}_{2}\sum _{j=1}^{N}{\dot{{d}_{j}}}^{2}{w}_{{A}_{2}}(\delta {t}_{j})\\ &amp; &amp; -{B}_{1}\sum _{j=1}^{N}\sum _{k=1}^{j-1}\dot{{d}_{k}}\dot{{d}_{j}}{w}_{{B}_{1}}({\mathrm{\Delta }}{t}_{{jk}},\delta {t}_{k},\delta {t}_{j})-{B}_{2}\sum _{j=1}^{N}\sum _{k=1}^{j-1}\dot{{d}_{k}}\dot{{d}_{j}}{w}_{{B}_{2}}({\mathrm{\Delta }}{t}_{{jk}},\delta {t}_{k},\delta {t}_{j})\end{array}\end{eqnarray*}

\begin{eqnarray*}{A}_{i}=\displaystyle \frac{1}{1+c}\cdot \displaystyle \frac{2}{{}^{\alpha }/\beta \cdot {\mu }_{i}},\qquad i\in \{1,2\}\end{eqnarray*}

\begin{eqnarray*}{B}_{i}=\displaystyle \frac{1}{1+c}\cdot \displaystyle \frac{2}{{}^{\alpha }/\beta \cdot {\mu }_{i}^{2}},\qquad i\in \{1,2\}\end{eqnarray*}

\begin{eqnarray*}{w}_{{A}_{i}}=\delta t-\displaystyle \frac{1}{{\mu }_{i}}\left(1-{e}^{-{\mu }_{i}\delta t}\right),\qquad i\in \{1,2\}\end{eqnarray*}

\begin{eqnarray*}{w}_{{B}_{i},{jk}}={e}^{-{\mu }_{i}{\mathrm{\Delta }}{t}_{{jk}}}({e}^{{\mu }_{i}\delta {t}_{k}}-1)({e}^{-{\mu }_{i}\delta {t}_{j}}-1),\qquad i\in \{1,2\}\end{eqnarray*}

\begin{eqnarray*}{\mathrm{\Delta }}{t}_{{jk}}={t}_{j}-{t}_{k}=\sum _{l=k}^{j-1}(\delta {t}_{l}+{g}_{l}).\end{eqnarray*}

#### C.4.2. Application of the dual envelopes

The dual envelope approach described in appendix C.1.2 is applied to the nonlinear terms ${w}_{{A}_{\mathrm{1,2}}},{w}_{{B}_{\mathrm{1,2}}}$. For simplicity and readability, we will limit the formal description to a single term of *w*_*A*_ and *w*_*B*_. The implementation for the individual repair-rates *μ*_1_, *μ*_2_, iso-centres, and possible interaction terms is completely analogous. For the interaction terms, the individual beam-on times are designated as *δ* *t*_*c*_ (the *current* iso-centre) and *δ* *t*_*p*_ (a *previously* delivered iso-centre).

The nonlinear function *w*_*A*_ is replaced with the convex envelope ${\hat{w}}_{A}$ constructed from the lower and upper bounds of the beam-on time $\underline{\delta t},\overline{\delta t}$.

\begin{eqnarray*}{\hat{w}}_{A}=\sum _{i\leqslant {2}^{k}}{\lambda }_{i}\prod _{j\leqslant k}{b}_{j}({d}_{{ij}})\end{eqnarray*}

\begin{eqnarray*}\forall i\leqslant {2}^{k},j\leqslant k\qquad {d}_{{ij}}=\left(\left\lfloor \displaystyle \frac{i-1}{{2}^{k-j}}\right\rfloor \mathrm{mod}2\right)\end{eqnarray*}

\begin{eqnarray*}\forall j\leqslant k\qquad {b}_{j}(0)={x}_{j}^{L}={w}_{A}(\underline{\delta t})\qquad \qquad \wedge \qquad \qquad {b}_{j}(1)={x}_{j}^{U}={w}_{A}(\overline{\delta t})\end{eqnarray*}

\begin{eqnarray*}\sum _{i\leqslant {2}^{k}}{\lambda }_{i}=1\qquad \qquad \wedge \qquad \qquad \forall i\leqslant {2}^{k},{\lambda }_{i}\geqslant 0.\end{eqnarray*}

Practically, since *k* = 1 for our application (single variable, *w*_*A*_ = ∏_*j*≤*k*_ *x*_*j*_), this boils down to a substitution of the nonlinear function *w*_*A*_ where the Lagrange multipliers *λ*_*i*_ act as a scaling factor within the bounded interval $[{w}_{A}(\underline{\delta t}),{w}_{A}(\overline{\delta t})]$. If, for example, we would add the dose-rate $\dot{d}$ as a decision variable, then this could easily be formulated as a bilinear function (*w*_*A*_ = *x*_1_ · *x*_2_) and the dual envelope would act as described in appendix C.1.2.

Similarly, the interaction term between the iso-centres *w*_*B*_ is a nonlinear function of three variables that require a convex relaxation to create a linear problem. First, we substitute the individual terms in the product to express *w*_*B*_ as a trilinear function:

\begin{eqnarray*}{w}_{B}={e}^{-\mu {\mathrm{\Delta }}{t}_{{cp}}}({e}^{\mu \delta {t}_{p}}-1)({e}^{-\mu \delta {t}_{c}}-1)={x}_{1}\cdot {x}_{2}\cdot {x}_{3}\end{eqnarray*}

\begin{eqnarray*}{x}_{1}={e}^{-\mu {\mathrm{\Delta }}{t}_{{cp}}},\qquad {x}_{2}={e}^{\mu \delta {t}_{p}}-1,\qquad {x}_{3}={e}^{-\mu \delta {t}_{c}}-1.\end{eqnarray*}

The bounds on the individual *x*_*j*_ are determined from the lower and upper bounds of the individual variables $\underline{{\mathrm{\Delta }}t},\overline{{\mathrm{\Delta }}t},\underline{\delta t},\overline{\delta t}$.

\begin{eqnarray*}{x}_{1}^{L}={e}^{-\mu \underline{{\mathrm{\Delta }}{t}_{{cp}}}},\qquad \qquad {x}_{2}^{L}={e}^{\mu \underline{\delta {t}_{p}}}-1,\qquad \qquad {x}_{3}^{L}={e}^{-\mu \underline{\delta {t}_{c}}}-1\end{eqnarray*}

\begin{eqnarray*}{x}_{1}^{U}={e}^{-\mu \overline{{\mathrm{\Delta }}{t}_{{cp}}}},\qquad \quad {x}_{2}^{U}={e}^{\mu \overline{\delta {t}_{p}}}-1,\qquad \qquad {x}_{3}^{U}={e}^{-\mu \overline{\delta {t}_{c}}}-1.\end{eqnarray*}

With this information, the dual envelop ${\hat{w}}_{B}$ of the trilinear interaction term (*k* = 3) is defined as follows:

\begin{eqnarray*}{\hat{w}}_{B}=\sum _{i\leqslant {2}^{k}}{\lambda }_{i}\prod _{j\leqslant k}{b}_{j}({d}_{{ij}})\end{eqnarray*}

\begin{eqnarray*}\forall j\leqslant k\qquad {x}_{j}=\sum _{i\leqslant {2}^{k}}{\lambda }_{i}{b}_{j}({d}_{{ij}})\end{eqnarray*}

\begin{eqnarray*}\forall i\leqslant {2}^{k},j\leqslant k\qquad {d}_{{ij}}=\left(\left\lfloor \displaystyle \frac{i-1}{{2}^{k-j}}\right\rfloor \mathrm{mod}2\right)\end{eqnarray*}

\begin{eqnarray*}\forall j\leqslant k\qquad {b}_{j}(0)={x}_{j}^{L}\qquad \qquad \wedge \qquad \qquad {b}_{j}(1)={x}_{j}^{U}\end{eqnarray*}

\begin{eqnarray*}\sum _{i\leqslant {2}^{k}}{\lambda }_{i}=1\qquad \qquad \wedge \qquad \qquad \forall i\leqslant {2}^{k},{\lambda }_{i}\geqslant 0.\end{eqnarray*}

Now the convex relaxation of the BED can be expressed with the beam-on times *δ* ***t*** and Lagrange multipliers *λ* as variables:

\begin{eqnarray*}\displaystyle \begin{array}{rcl}\hat{{BED}} &amp; = &amp; \sum _{j=1}^{N}\dot{{d}_{j}}\delta {t}_{j}+{A}_{1}\sum _{j=1}^{N}{\dot{{d}_{j}}}^{2}{\hat{w}}_{{A}_{1},j}+{A}_{2}\sum _{j=1}^{N}{\dot{{d}_{j}}}^{2}{\hat{w}}_{{A}_{2},j}\\ &amp; &amp; -{B}_{1}\sum _{j=1}^{N}\sum _{k=1}^{j-1}\dot{{d}_{k}}\dot{{d}_{j}}{\hat{w}}_{{B}_{1},{jk}}-{B}_{2}\sum _{j=1}^{N}\sum _{k=1}^{j-1}\dot{{d}_{k}}\dot{{d}_{j}}{\hat{w}}_{{B}_{2},{jk}}.\end{array}\end{eqnarray*}

#### C.4.3. Application of PWLs

Up until now, all terms in the convex BED formulation are completely independent of each other. However, in practice, each individual iso-centre will contribute to multiple of these terms For example, iso-centre *j* will interact with all previously delivered iso-centres *k* < *j* in one way and all following iso-centres *k* > *j* in another way (see *x*_1_, *x*_2_, *x*_3_ in equation (54)). Thus, we must enforce consistency for all the timing information across the individual envelopes. Since the individual terms substituted in the dual envelope approach are bijective over the domain ${{\mathbb{R}}}_{\geqslant 0}$ of the timing variables (*δ* *t* and Δ*t*) and their corresponding codomains, they can be inverted. Thus, one can retrieve the timing information from the state of the variables of the dual envelopes. To not re-introduce nonlinearities into our system, we apply PWLs to these nonlinear terms to determine the values of the timing variables *δ* *t* and Δ*t*.

By enforcing an equality constraint between the PWL of the nonlinear terms *L* and the corresponding values from the dual envelope (${x}_{j},\hat{w}$), the timing variables can be extracted from the current state of the PWL.

For ${\hat{w}}_{A}$ the relationship is as follows:

\begin{eqnarray*}L\left(\delta t-\displaystyle \frac{1}{\mu }\left(1-{e}^{-\mu \delta t}\right)\right)=\sum _{l=0}^{m}\left({a}_{l}-\displaystyle \frac{1}{\mu }\left(1-{e}^{-\mu {a}_{l}}\right)\right){\nu }_{l}=\sum _{i\leqslant {2}^{k}}{\lambda }_{i}{b}_{1}({d}_{i1})\end{eqnarray*}

\begin{eqnarray*}\Rightarrow \delta t=\sum _{l=0}^{m}{a}_{l}{\nu }_{l}.\end{eqnarray*}

For ${\hat{w}}_{B}$ we need to create a PWL for each of the three terms (*x*_1_, *x*_2_, *x*_3_) and enforce the same equality constraints:

\begin{eqnarray*}L({x}_{j})=\sum _{l=0}^{m}{x}_{j}({a}_{l}){\nu }_{l}=\sum _{i\leqslant {2}^{k}}{\lambda }_{i}{b}_{j}({d}_{{ij}}),\qquad j\in \{1,2,3\},k=3\end{eqnarray*}

\begin{eqnarray*}j=1:L\left({e}^{-\mu {\mathrm{\Delta }}{t}_{{cp}}}\right)=\sum _{l=0}^{m}\left({e}^{-\mu {a}_{l}}\right){\nu }_{l}=\sum _{i\leqslant {2}^{k}}{\lambda }_{i}{b}_{1}({d}_{i1})\Rightarrow {\mathrm{\Delta }}{t}_{{cp}}=\sum _{l=0}^{m}{a}_{l}{\nu }_{l}\end{eqnarray*}

\begin{eqnarray*}j=2:L\left({e}^{\mu \delta {t}_{p}}-1\right)=\sum _{l=0}^{m}\left({e}^{\mu {a}_{l}}-1\right){\nu }_{l}\,=\sum _{i\leqslant {2}^{k}}{\lambda }_{i}{b}_{2}({d}_{i2})\Rightarrow \delta {t}_{p}=\sum _{l=0}^{m}{a}_{l}{\nu }_{l}\end{eqnarray*}

\begin{eqnarray*}j=3:L\left({e}^{-\mu \delta {t}_{c}}-1\right)=\sum _{l=0}^{m}\left({e}^{-\mu {a}_{l}}-1\right){\nu }_{l}\,=\sum _{i\leqslant {2}^{k}}{\lambda }_{i}{b}_{3}({d}_{i3})\Rightarrow \delta {t}_{c}=\sum _{l=0}^{m}{a}_{l}{\nu }_{l}.\end{eqnarray*}

As mentioned above, these equations are exemplary for individual envelopes. The introduced constraints need to be enforced across all terms in the BED formulation. For each of the two repair-rates there are *N*_*iso*_ ${\hat{w}}_{A}$'s and ${N}_{{ia}}=\tfrac{{N}_{{iso}}({N}_{{iso}}-1)}{2}$ ${\hat{w}}_{B}$'s. With the constraints on every individual term, we can ensure that the timing of the iso-centres is consistent across all envelopes even after switching to the Lagrange multipliers as the variables.

#### C.4.4. Objective function

To implement the objective function (see equation (6)), a new cost variable is introduced:

\begin{eqnarray*}f(\hat{{\bf{BED}}})={w}_{TV}\displaystyle \sum _{v\in TV}\frac{{{cost}}_{v}}{{N}_{TV}}+{w}_{Rim}\displaystyle \sum _{v\in Rim}\frac{{{cost}}_{v}}{{N}_{Rim}}.\end{eqnarray*}

Using inequality constraints for every voxel *v*, it can be ensured that only BED values beyond the defined lower BED_*ref*_ and upper bounds BED_*thres*_ contribute to the penalty value (i.e. no negative penalty is incurred for voxels within their defined bounds)

\begin{eqnarray*}{{cost}}_{v}\geqslant \left\{\begin{array}{ll}{\mathrm{BED}}_{{ref}}-{\hat{\mathrm{BED}}}_{v} &amp; \forall v\in TV\\ {\hat{\mathrm{BED}}}_{v}-{\mathrm{BED}}_{{thres}} &amp; \forall v\in Rim\end{array}\right..\end{eqnarray*}

#### C.4.5. Beam-on time MILP problem

With the approaches introduced above, we can now define the beam-on time optimisation as a MILP problem. The parameters controlling the number of support points used for the PWLs (*m*_*A*_ and *m*_*B*,1_, *m*_*B*,2_, *m*_*B*,3_) allow for a trade-off between the accuracy of the approximations and the incurred computational cost. An appropriate choice of this parameter becomes crucial when dealing with the sequence optimisation (see appendix C.5.1). For the beam-on time MILP problem, two support points were used for every envelope.

\begin{eqnarray*}\begin{array}{l}\begin{array}{llll}\mathrm{minimise} &amp; f({\bf{B}}\hat{{\bf{E}}}{\bf{D}}) &amp; &amp; \\ {\mathrm{w}}.{\mathrm{r}}.{\mathrm{t}}. &amp; \delta {\boldsymbol{t}}\in {{\mathbb{R}}}_{\geqslant 0}^{{N}_{{iso}}} &amp; {\mathrm{\Delta }}{\boldsymbol{t}}\in {{\mathbb{R}}}_{\geqslant 0}^{{N}_{{ia}}} &amp; \\ &amp; {\hat{{\boldsymbol{w}}}}^{A}\in {{\mathbb{R}}}_{\geqslant 0}^{2\times {N}_{{iso}}} &amp; {\lambda }^{A}\in {{\mathbb{R}}}_{\geqslant 0}^{2\times {N}_{{iso}}\times 2} &amp; \\ &amp; {\boldsymbol{L}}({w}^{A})\in {{\mathbb{R}}}_{\geqslant 0}^{2\times {N}_{{iso}}} &amp; {\nu }^{A}\in {{\mathbb{R}}}_{\geqslant 0}^{2\times {N}_{{iso}}\times {m}_{A}} &amp; {{\boldsymbol{y}}}^{A}\in \{0,1\}{}^{2\times {N}_{{iso}}\times {m}_{A}-1}\\ &amp; {\hat{{\boldsymbol{w}}}}^{B}\in {{\mathbb{R}}}_{\leqslant 0}^{2\times {N}_{{ia}}} &amp; {\lambda }^{B}\in {{\mathbb{R}}}_{\geqslant 0}^{2\times {N}_{{ia}}\times 8} &amp; \\ &amp; {{\boldsymbol{x}}}_{1}^{B}\in {{\mathbb{R}}}_{\geqslant 0}^{2\times {N}_{{ia}}} &amp; {{\boldsymbol{x}}}_{2}^{B}\in {{\mathbb{R}}}_{\geqslant 0}^{2\times {N}_{{iso}}} &amp; {{\boldsymbol{x}}}_{3}^{B}\in {{\mathbb{R}}}_{\leqslant 0}^{2\times {N}_{{iso}}}\\ &amp; {\boldsymbol{L}}({{\boldsymbol{x}}}_{1}^{B})\in {{\mathbb{R}}}_{\geqslant 0}^{2\times {N}_{{ia}}} &amp; {\nu }_{1}^{B}\in {{\mathbb{R}}}_{\geqslant 0}^{2\times {N}_{{ia}}\times {m}_{B,1}} &amp; {{\boldsymbol{y}}}_{1}^{B}\in \{0,1\}{}^{2\times {N}_{{ia}}\times {m}_{B,1}-1}\\ &amp; {\boldsymbol{L}}({{\boldsymbol{x}}}_{2}^{B})\in {{\mathbb{R}}}_{\geqslant 0}^{2\times {N}_{{iso}}} &amp; {\nu }_{2}^{B}\in {{\mathbb{R}}}_{\geqslant 0}^{2\times {N}_{{iso}}\times {m}_{B,2}} &amp; {{\boldsymbol{y}}}_{2}^{B}\in \{0,1\}{}^{2\times {N}_{{iso}}\times {m}_{B,2}-1}\\ &amp; {\boldsymbol{L}}({{\boldsymbol{x}}}_{3}^{B})\in {{\mathbb{R}}}_{\leqslant 0}^{2\times {N}_{{iso}}} &amp; {\nu }_{3}^{B}\in {{\mathbb{R}}}_{\geqslant 0}^{2\times {N}_{{iso}}\times {m}_{B,3}} &amp; {{\boldsymbol{y}}}_{3}^{B}\in \{0,1\}{}^{2\times {N}_{{iso}}\times {m}_{B,3}-1}\\ &amp; {\boldsymbol{c}}{{ost}}_{i}\in {{\mathbb{R}}}_{\geqslant 0}^{{N}_{{vox}}} &amp; &amp; \end{array}\end{array}\end{eqnarray*}

\begin{eqnarray*}\begin{array}{cccc}{\mathrm{s}}.{\mathrm{t}}. &amp; (49)-(52) &amp; (57)-(61) &amp; (\mathrm{dual}\,\mathrm{env}.\,\mathrm{constr}.)\\ &amp; (34)-(40) &amp; (63)-(68) &amp; (\mathrm{PWL}\,\mathrm{constr}.)\\ &amp; (62) &amp; (69)-(70) &amp; (\mathrm{cost}\,\mathrm{constr}.).\end{array}\end{eqnarray*}

#### C.4.6. Adding sequence optimisation

To add the ability to change the delivery sequence, we introduce a square matrix variable **h** ∈ {0, 1}^*N*×*N*^ controlling the active terms

\begin{eqnarray*}\begin{array}{rcl}\hat{\mathrm{BED}} &amp; = &amp; \displaystyle \sum _{j=1}^{N}\dot{{d}_{j}}\delta {t}_{j}+{A}_{1}\displaystyle \sum _{j=1}^{N}{\dot{{d}_{j}}}^{2}{\hat{w}}_{{A}_{1},j}+{A}_{2}\displaystyle \sum _{j=1}^{N}{\dot{{d}_{j}}}^{2}{\hat{w}}_{{A}_{2},j}\\ &amp; &amp; -{B}_{1}\displaystyle \sum _{j\ne k}{h}_{{jk}}\dot{{d}_{k}}\dot{{d}_{j}}{\hat{w}}_{{B}_{1},{jk}}-{B}_{2}\displaystyle \sum _{j\ne k}{h}_{{jk}}\dot{{d}_{k}}\dot{{d}_{j}}{\hat{w}}_{{B}_{2},{jk}}\end{array}\end{eqnarray*}

and enforce a constraint that limits the active terms to the physically possible ones. That is: there are always $\tfrac{N(N-1)}{2}$ active terms and only one active interaction term for any combination of two iso-centres *j*, *k*.

\begin{eqnarray*}\forall j,k\in \{1,\ldots ,N\},j\ne k\qquad \qquad \qquad {h}_{{jk}}+{h}_{{kj}}=1.\end{eqnarray*}

Now the solver would be able to activate and deactivate all the individual interaction terms. However, when changing the sequence of delivery there is more changing than only the active interaction terms The time between two iso-centres depends on the beam-on times of all the iso-centres delivered in-between them. Thus, we need to be able to relate the active terms, to the delivery sequence. To that end we introduce the matrix **s** ∈ {0, 1}^*N*×*N*^, which describes which iso-centre is delivered at which position in the order of delivery. The column describes the position in the sequence and the non-zero row index determines the iso-centre used in that position

\begin{eqnarray*}{\bf{seq}}=\left[\begin{array}{ccc}2 &amp; 1 &amp; 3\end{array}\right]\iff {\bf{s}}=\left[\begin{array}{ccc}0 &amp; 1 &amp; 0\\ 1 &amp; 0 &amp; 0\\ 0 &amp; 0 &amp; 1\end{array}\right]\iff {\bf{h}}=\left[\begin{array}{ccc}- &amp; 1 &amp; 0\\ 0 &amp; - &amp; 0\\ 1 &amp; 1 &amp; -\end{array}\right].\end{eqnarray*}

Again, we need to ensure that **s** is constrained to a feasible treatment, i.e. exactly one iso-centre delivered at every position (column) and each iso-centre (row index) must be used once

\begin{eqnarray*}\forall i\in \{1,\ldots ,N\},\qquad \qquad \sum _{j=1}^{N}{s}_{{ij}}=1\qquad \qquad \wedge \qquad \qquad \sum _{j=1}^{N}{s}_{{ij}}=1.\end{eqnarray*}

To enforce the consistency between the variables for iso-centre order **s** and the active terms **h**, we introduce a comparison function *comp*_*jk*_ between two iso-centres *j* and *k*:

\begin{eqnarray*}{{comp}}_{{jk}}=\sum _{i=1}^{N-1}(N-i)({s}_{{ij}}-{s}_{{ik}}).\end{eqnarray*}

*comp*_*jk*_ ∈ { − (*N* −1),…, −1, 1,…,* N* − 1} will be positive if iso-centre *j* is delivered before iso-centre *k* (i.e. *t*_*j*_ < *t*_*k*_), and negative if iso-centre *j* is delivered after iso-centre *k* (i.e. *t*_*j*_ > *t*_*k*_). Thus, the binary interaction term control variable *h*_*jk*_ needs to be one for all combinations of *j* and *k* where *comp*_*jk*_ < 0. This can be enforced with the following inequality constraint:

\begin{eqnarray*}\forall j,k\in \{1,\ldots ,N\},j\ne k\qquad \qquad {h}_{{jk}}\cdot {{comp}}_{{jk}}+{h}_{{kj}}\cdot {{comp}}_{{kj}}\le -1.\end{eqnarray*}

Now that the active terms **h** directly represent the current sequence, the starting time of every iso-centre *j* can be defined as follows:

\begin{eqnarray*}{t}_{j}=\sum _{k=1,k\ne j}^{N}{h}_{{jk}}\left(\delta {t}_{k}+{g}_{k}\right).\end{eqnarray*}

We can now use this starting time definition to constrain the value of Δ*t*_*jk*_ to the appropriate value based on the delivery sequence:

\begin{eqnarray*}{h}_{{jk}}{\mathrm{\Delta }}{t}_{{jk}}={h}_{{jk}}({t}_{j}-{t}_{k}).\end{eqnarray*}

The use of the binary variable *h*_*jk*_ is required here to ensure compatibility with the dual envelopes and PWLs which only allow values of Δ*t*_*jk*_ within the predefined bounds $\left[\underline{{\mathrm{\Delta }}{t}_{{jk}}},\overline{{\mathrm{\Delta }}{t}_{{jk}}}\right]$ where $\underline{{\mathrm{\Delta }}{t}_{{jk}}}> 0$.

At this point, the problem fully is described and the convex relaxation of the BED could be optimised. However, with the additional binary variables introduced above, the problem is no longer linear. There are multiplications of the binary variable **h** with the interaction terms ${\hat{w}}_{B}$, sequence **s**, beam-on times *δ* *t*, starting times *t*, and time in-between iso-centre deliveries Δ*t*. Every one if these nonlinearities is resolved using the big *M* method described in appendix C.2. The required inequalities are completely analogous to that description and are omitted here for clarity:

\begin{eqnarray*}\forall j,k\in \{1,\ldots ,N\},j\ne k{\mathrm{:}}\,\end{eqnarray*}

\begin{eqnarray*}\begin{array}{rrcc}\forall i\in \{1,2\}: &amp; \,{h}_{{jk}}{\hat{w}}_{{B}_{i},{jk}} &amp; \Rightarrow &amp; {\hat{z}}_{{jk}}^{{B}_{i}}\end{array}\end{eqnarray*}

\begin{eqnarray*}\begin{array}{lccc}\forall i\in \{1,\ldots ,N-1\},l=j\vee l=k:\, &amp; {h}_{{jk}}{s}_{{il}} &amp; \Rightarrow &amp; {z}_{{jk},{il}}^{S}\end{array}\end{eqnarray*}

\begin{eqnarray*}\,\begin{array}{crcc} &amp; {h}_{{jk}}\delta {t}_{k} &amp; \Rightarrow &amp; {z}_{{jk}}^{\delta t}\end{array}\end{eqnarray*}

\begin{eqnarray*}\,\begin{array}{cccc} &amp; {h}_{{jk}}{t}_{j} &amp; \Rightarrow &amp; {z}_{{jk}}^{{T}^{+}}\end{array}\end{eqnarray*}

\begin{eqnarray*}\,\begin{array}{cccc} &amp; {h}_{{jk}}{t}_{k} &amp; \Rightarrow &amp; {z}_{{jk}}^{{T}^{-}}\end{array}\end{eqnarray*}

\begin{eqnarray*}\,\begin{array}{cccc} &amp; {h}_{{jk}}{\mathrm{\Delta }}{t}_{{jk}} &amp; \Rightarrow &amp; {z}_{{jk}}^{{\mathrm{\Delta }}t}.\end{array}\end{eqnarray*}

Now the convex relaxation of the BED for the simultaneous beam-on time and sequence optimisation can be defined as follows:

\begin{eqnarray*}\begin{array}{rcl}\hat{\mathrm{BED}} &amp; = &amp; \displaystyle \sum _{j=1}^{N}\dot{{d}_{j}}\delta {t}_{j}+{A}_{1}\displaystyle \sum _{j=1}^{N}{\dot{{d}_{j}}}^{2}{\hat{w}}_{{A}_{1},j}+{A}_{2}\displaystyle \sum _{j=1}^{N}{\dot{{d}_{j}}}^{2}{\hat{w}}_{{A}_{2},j}\\ &amp; &amp; -{B}_{1}\displaystyle \sum _{j\ne k}\dot{{d}_{k}}\dot{{d}_{j}}{\hat{z}}_{{jk}}^{{B}_{1}}-{B}_{2}\displaystyle \sum _{j\ne k}\dot{{d}_{k}}\dot{{d}_{j}}{\hat{z}}_{{jk}}^{{B}_{2}}\end{array}\end{eqnarray*}

### C.5. Beam-on time and sequence MILP problem

With the variables and constraints introduced above, we can now formulate a MILP problem for the beam-on time and sequence optimisation.

\begin{eqnarray*}\begin{array}{l}\begin{array}{llll}\mathrm{minimise} &amp; f({\bf{B}}\hat{{\bf{E}}}{\bf{D}}) &amp; &amp; \\ {\mathrm{w}}.{\mathrm{r}}.{\mathrm{t}}. &amp; {\boldsymbol{s}}\in \{0,1\}{}^{{N}_{{iso}}\times {N}_{{iso}}} &amp; {\boldsymbol{h}}\in \{0,1\}{}^{{N}_{{iso}}\times {N}_{{iso}}} &amp; {{\boldsymbol{z}}}^{S}\in \{0,1\}{}^{{N}_{{iso}}\times {N}_{{iso}}\times {N}_{{iso}}-1\times {N}_{{iso}}}\\ &amp; \delta {\boldsymbol{t}}\in {{\mathbb{R}}}_{\geqslant 0}^{{N}_{{iso}}} &amp; {\boldsymbol{t}}\in {{\mathbb{R}}}_{\geqslant 0}^{{N}_{{iso}}} &amp; {\mathrm{\Delta }}{\boldsymbol{t}}\in {{\mathbb{R}}}_{\geqslant 0}^{{N}_{{iso}}\times {N}_{{iso}}}\\ &amp; {{\boldsymbol{z}}}^{\delta t}\in {{\mathbb{R}}}_{\geqslant 0}^{{N}_{{iso}}\times {N}_{{iso}}} &amp; {{\boldsymbol{z}}}^{{T}^{+}}\in {{\mathbb{R}}}_{\geqslant 0}^{{N}_{{iso}}\times {N}_{{iso}}} &amp; {{\boldsymbol{z}}}^{{T}^{-}}\in {{\mathbb{R}}}_{\geqslant 0}^{{N}_{{iso}}\times {N}_{{iso}}}\\ &amp; {{\boldsymbol{z}}}^{{\mathrm{\Delta }}t}\in {{\mathbb{R}}}_{\geqslant 0}^{{N}_{{iso}}\times {N}_{{iso}}} &amp; {\hat{{\boldsymbol{z}}}}^{B}\in {{\mathbb{R}}}_{\geqslant 0}^{2\times {N}_{{iso}}\times {N}_{{iso}}} &amp; \\ &amp; {\hat{{\boldsymbol{w}}}}^{A}\in {{\mathbb{R}}}_{\geqslant 0}^{2\times {N}_{{iso}}} &amp; {\lambda }^{A}\in {{\mathbb{R}}}_{\geqslant 0}^{2\times {N}_{{iso}}\times 2} &amp; \\ &amp; {\boldsymbol{L}}({w}^{A})\in {{\mathbb{R}}}_{\geqslant 0}^{2\times {N}_{{iso}}} &amp; {\nu }^{A}\in {{\mathbb{R}}}_{\geqslant 0}^{2\times {N}_{{iso}}\times {m}_{A}} &amp; {{\boldsymbol{y}}}^{A}\in \{0,1\}{}^{2\times {N}_{{iso}}\times {m}_{A}-1}\\ &amp; {\hat{{\boldsymbol{w}}}}^{B}\in {{\mathbb{R}}}_{\leqslant 0}^{2\times {N}_{{iso}}\times {N}_{{iso}}} &amp; {\lambda }^{B}\in {{\mathbb{R}}}_{\geqslant 0}^{2\times {N}_{{iso}}\times {N}_{{iso}}\times 8} &amp; \\ &amp; {{\boldsymbol{x}}}_{1}^{B}\in {{\mathbb{R}}}_{\geqslant 0}^{2\times {N}_{{iso}}\times {N}_{{iso}}} &amp; {{\boldsymbol{x}}}_{2}^{B}\in {{\mathbb{R}}}_{\geqslant 0}^{2\times {N}_{{iso}}} &amp; {{\boldsymbol{x}}}_{3}^{B}\in {{\mathbb{R}}}_{\leqslant 0}^{2\times {N}_{{iso}}}\\ &amp; {\boldsymbol{L}}({{\boldsymbol{x}}}_{1}^{B})\in {{\mathbb{R}}}_{\geqslant 0}^{2\times {N}_{{iso}}\times {N}_{{iso}}} &amp; {\nu }_{1}^{B}\in {{\mathbb{R}}}_{\geqslant 0}^{2\times {N}_{{iso}}\times {N}_{{iso}}\times {m}_{B,1}} &amp; {{\boldsymbol{y}}}_{1}^{B}\in \{0,1\}{}^{2\times {N}_{{iso}}\times {N}_{{iso}}\times {m}_{B,1}-1}\\ &amp; {\boldsymbol{L}}({{\boldsymbol{x}}}_{2}^{B})\in {{\mathbb{R}}}_{\geqslant 0}^{2\times {N}_{{iso}}} &amp; {\nu }_{2}^{B}\in {{\mathbb{R}}}_{\geqslant 0}^{2\times {N}_{{iso}}\times {m}_{B,2}} &amp; {{\boldsymbol{y}}}_{2}^{B}\in \{0,1\}{}^{2\times {N}_{{iso}}\times {m}_{B,2}-1}\\ &amp; {\boldsymbol{L}}({{\boldsymbol{x}}}_{3}^{B})\in {{\mathbb{R}}}_{\leqslant 0}^{2\times {N}_{{iso}}} &amp; {\nu }_{3}^{B}\in {{\mathbb{R}}}_{\geqslant 0}^{2\times {N}_{{iso}}\times {m}_{B,3}} &amp; {{\boldsymbol{y}}}_{3}^{B}\in \{0,1\}{}^{2\times {N}_{{iso}}\times {m}_{B,3}-1}\\ &amp; {\boldsymbol{c}}{{ost}}_{i}\in {{\mathbb{R}}}_{\geqslant 0}^{{N}_{{vox}}} &amp; &amp; \end{array}\end{array}\end{eqnarray*}

\begin{eqnarray*}\begin{array}{cccc}{\mathrm{s}}.{\mathrm{t}}. &amp; (73) &amp; (75)-(79) &amp; (\mathrm{seq}.\,\mathrm{constr}.)\\ &amp; (23)-(33) &amp; (80)-(85) &amp; (\mathrm{big}\,M\,\mathrm{constr}.)\\ &amp; (49)-(52) &amp; (57)-(61) &amp; (\mathrm{dual}\,\mathrm{env}.\,\mathrm{constr}.)\\ &amp; (34)-(40) &amp; (63)-(68) &amp; (\mathrm{PWL}\,\mathrm{constr}.)\\ &amp; (69)-(70) &amp; (86) &amp; (\mathrm{cost}\,\mathrm{constr}.).\end{array}\end{eqnarray*}

*Note:* All variables that were specific to the ${N}_{{ia}}=\tfrac{{N}_{{iso}}({N}_{{iso}}-1)}{2}$ interaction terms in equation (71) are now expressed with *N*_*iso*_ × *N*_*iso*_ individual components to cover all possible interactions. Their diagonal elements (i.e. ‘self-interaction’ of an iso-centre) do not contribute to the optimisation problem.

#### C.5.1. PWL considerations for the sequence optimisation

As described above, to solve the treatment planning problem the convex relaxation of the BED is built from a set of bounds on the iso-centre timings (*δ* *t*, Δ*t*). For the beam-on time optimisation, the bounds of Δ*t* follow directly from the bounds on the individual beam-on times. As the bounds on beam-on times tighten, so do the bounds on the possible Δ*t*. Thus, the convex relaxation of the BED converges to the value of the full nonlinear version even if we only use the minimum of two support points in the PWLs. This substantially reduces the computational cost of solving the model.

When the order of iso-centre delivery can change, then the range of possible values for Δ*t* will not shrink with the bounds on the beam-on time. Instead, its lower limit is determined by the shortest possible beam-on time and the upper limit is defined by the (*N* − 1) longest possible beam-on times. In order to ensure accurate values for Δ*t* (and thus the BED) are determined from the PWL of the corresponding nonlinear term, the number of support points needs to be increased. Preliminary tests determined that using 10 support points was sufficient to accurately determine the BED from the convex relaxation.
